# A Polyproline Type II Peptidomimetic Disrupts a Grb2 SH3C Domain Protein–Protein Interaction Implicated in Breast Cancer

**DOI:** 10.1002/cbic.202500343

**Published:** 2025-06-06

**Authors:** James Luccarelli, Philip C. Simister, Andrew D. Hamilton, Stephan M. Feller, Sam Thompson

**Affiliations:** ^1^ Chemistry Research Laboratory Department of Chemistry University of Oxford 12 Mansfield Road Oxford OX1 3TA UK; ^2^ Biological Systems Architecture Group Weatherall Institute of Molecular Medicine University of Oxford Oxford OX3 9DS UK; ^3^ Department of Chemistry New York University 100 Washington Square East New York NY 10003 USA; ^4^ Institute for Molecular Medicine Martin‐Luther University Halle‐Wittenberg 06120 Halle Saale Germany; ^5^ School of Chemistry and The Institute for Life Sciences University of Southampton Southampton SO17 1BJ UK

**Keywords:** Her2, mimic, protein–protein interactions, secondary structures, SH3 domains

## Abstract

Given the essential role of protein–protein interactions (PPIs) in cellular signaling pathways, their selective modulation is of great therapeutic interest. Mimicry of secondary structural protein elements has emerged as a promising strategy, with various scaffolds reproducing recognition surfaces of *α*–helical and *β*–strand/sheet proteins. A critical PPI, controlling cell growth and proliferation in breast and other cancers, occurs between growth factor receptor‐bound protein 2 (Grb2) and a polyproline II (PPII) helix embedded in Gab2. Herein, the first example of a general approach for nonpeptidic mimicry of extended PPII helices is presented and it is demonstrated that the scaffold may be functionalized to recapitulate the binding characteristics of crucial hydrophobic and cationic Gab2 hot‐spot side‐chains. The rationally designed peptidomimetic binds Grb2 at the same position as Gab2 (protein‐observed nuclear magnetic resonance (NMR)) with affinities comparable to the native peptide sequence (surface plasmon resonance (SPR)). With the addition of a new PPII minimalist scaffold, these studies further validate the use of diverse secondary structure peptidomimetics in disrupting therapeutically relevant PPIs.

## Graphical abstract

Benzoylurea scaffolds accurately mimic the side‐chain vectors of the i, i + 1/2, and i + 3 positions of polyproline type II helices, showing functional activity in the disruption of a protein–protein interaction implicated in breast cancer.

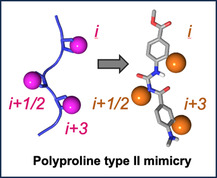

## 1 Introduction

Protein–protein interactions are key modulators of cellular states,^[^ ^1^ ^]^ and pharmacologic mediation of these interactions is a promising strategy for treatment of currently intractable human disease.^[^ ^2^ ^]^ Despite the urgent need for small molecules that can selectively modify protein–protein interactions (PPIs), the first molecule working by this mechanism was not FDA‐approved until 2016.^[^ ^3^ ^]^ Although PPI interaction surfaces are generally broad and lack distinct binding pockets, frequently clusters of amino acid side‐chains, or “hot‐spots” are crucial in determining selectivity and affinity for a cognate partner.^[^ ^4^, ^5^ ^]^ In many cases, these side‐chains are projected from a secondary structural protein element, prompting researchers to develop synthetic scaffolds to mimic the spatial and angular orientation of their side‐chain residues. Much work has been directed toward *α*–helical^[^ ^6^, ^7^, ^8^ ^]^ and *β*‐strand^[^ ^9^, ^10^, ^11^, ^12^ ^]^ mimics of this type, with constructs demonstrating efficacy against a range of biologic targets including islet amyloid polypeptide,^[^ ^13^, ^14^, ^15^ ^]^ HIF–1α/p300^[^ ^16^, ^17^ ^]^ and Bcl‐_X_L.^[^ ^18^, ^19^ ^]^ Despite these successes, there is a pressing need for an expanded toolkit of scaffolds to reproduce the diversity of secondary structures found at protein interaction surfaces.

**Figure 1:**
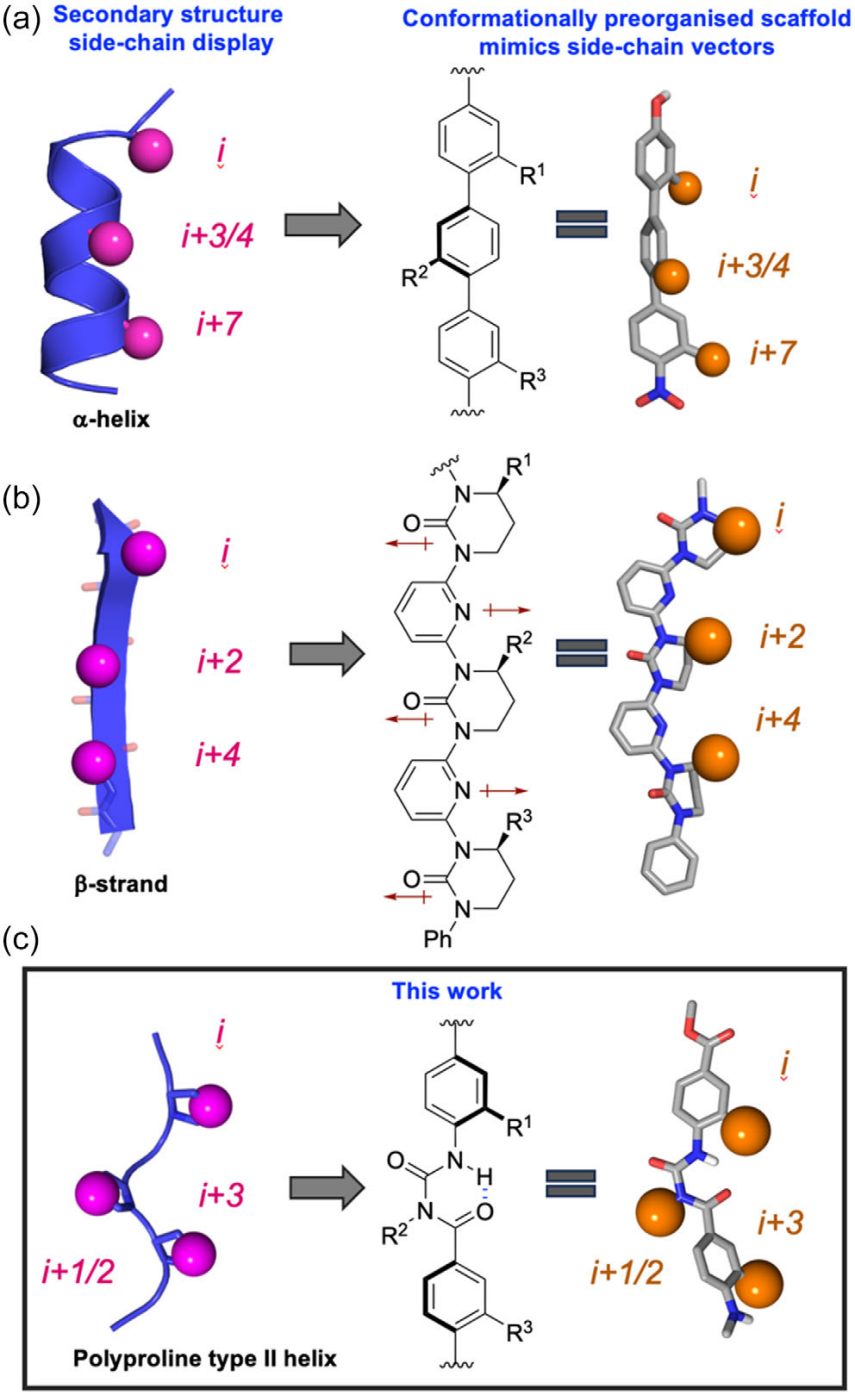
Rational design of nonpeptidic multivalent PPI mediators. Groups of side‐chains projected from one face of protein secondary structural elements constitute critical “hot‐spots” on the recognition surfaces. Canonical secondary structures with examples of their corresponding minimalist peptidomimetics; a) *α*‐helix, b) *β*‐strand, and c) PPII helix.

Extended polyproline type II (PPII) helices, structures that are heavily represented in intracellular signaling domains, have received comparatively little attention from a peptidomimetic perspective.^[^ ^20^, ^21^, ^22^ ^]^ Compared to the *α*‐helix, PPII helices are more extended (≈9 Å per turn vs 5.4 Å) and feature a threefold axis of symmetry (**Figure** **1**a,c). Current PPII mimics rely on derivatives of proline residues,^[^ ^23^, ^24^ ^]^ or the incorporation of structurally optimized Pro‐Pro dipeptide mimics.^[^ ^25^, ^26^, ^27^, ^28^, ^29^ ^]^

An important therapeutic target mediated by a PPII structure is that of the Gab2 multisite docking protein,^[^ ^30^ ^]^ which interacts with the C–terminal SH3 domain (SH3C) of Grb2 through two epitopes with Arg‐x‐x‐Lys (*x* = any residue) motifs embedded within PPII and 3_10_ helices.^[^ ^31^ ^]^ This interaction links cell‐surface receptors, such as Her2, to intracellular signaling cascades, contributing to cell growth and proliferation in breast cancer,^[^ ^32^ ^]^ acute lymphoblastic leukemia,^[^ ^33^ ^]^ and other malignancies.^[^ ^34^ ^]^ Truncates of the Gab2a/b peptides identified the strongest peptide binders of Grb2: a 20mer of Gab2a (GST‐tagged, residues 348–367; PPII conformation), and a 15mer of Gab2b (residues 508–522; mixed PPII/3_10_ conformation).^[^ ^31^ ^]^ To date, low‐affinity small molecules discovered through virtual screens have shown the ability to bind to Grb2 SH3C and to disrupt Gab2 binding, but more selective and potent inhibitors await development.^[^ ^35^, ^36^, ^37^ ^]^

*α*‐Helical peptidomimetics based on a benzoylurea foldamer^[^ ^38^, ^39^, ^40^ ^]^ have shown micromolar inhibition of the Bcl‐_*X*_L‐Bak interaction, while maintaining selectivity against other protein targets.^[^ ^18^, ^19^ ^]^ Given these successes, we hypothesized that this scaffold might be adapted to reproduce the positions and vectors of amphiphilic side‐chain combinations projected from the PPII helix of Gab2a, and thus provide a platform from which to optimize inhibition of the Grb2‐Gab2a PPI.

**Figure 2:**
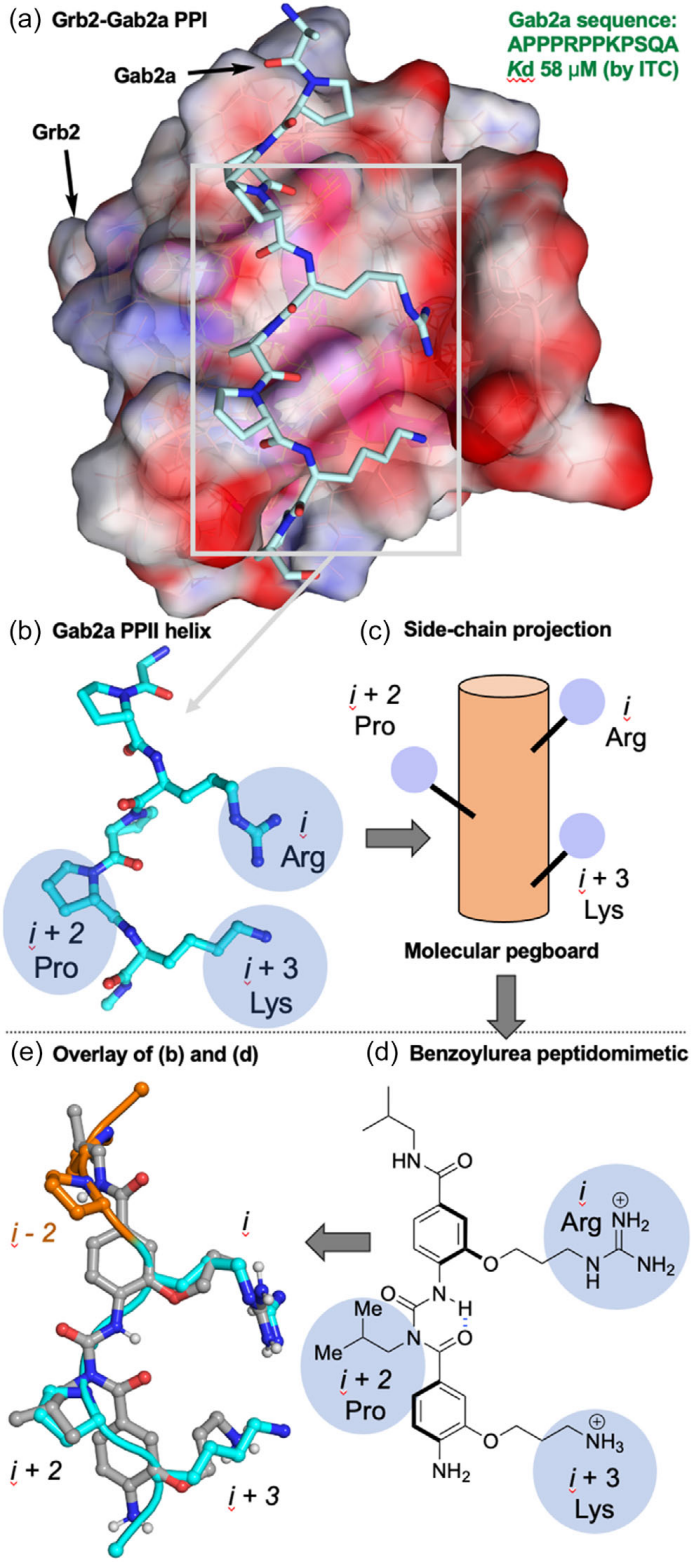
a) Grb2/Gab2a PPI (PDB 2W0Z), b) side‐chain projection from polyproline type II helix of Gab2a peptide, c) schematic side‐chain projection, d) benzoylurea peptidomimetic **1**, and e) superimposition of low energy conformation (see main text) of mimetic and Gab2a peptide, RMSD 0.52 Å for the Gab2a *C*_*α*_ and *C*_*β*_ positions of the *i*, *i + 2*, and *i + 3* side‐chains and the corresponding positions of mimetic **1**.

In contrast to mimicry of the *i*, *i* + 3/*i* + 4, and *i* + 7 residues along one face of an α‐helix, the scaffold reproduces the *i*, *i* + 1/*i* + 2, and *i* + 3 residues of the PPII helix (**Figure** 1c and **2**b,c). Thus, the target peptidomimetic **1** reproduces the positions and angular projection of the critical Arg354 (*i*) and Lys357 (*i + *3) residues of Gab2a, with the central isobutyl group providing a hydrophobic mimic of Pro356 (*i + *2) and the *C*‐terminal *iso*‐butyl amide potentially mimicking Pro352 (*i‐2* orange; Figure 2c/d). The highly flexible alkoxy groups, bearing the Arg and Lys mimics, make meaningful a priori modeling of global conformation challenging, especially in a competitive solvent such as water. However, there are extensive solid‐ and solution‐phase spectroscopic data of variously substituted benzoylureas, which provides a common conformational model for the backbone, the side‐chain vectors, and thus a basis for the atomic positions that mimic *C*_*α*_ and *C*_*β*_ for *i*, *i + 2*, and *i + 3* of Gab2a. The resulting conformational model of mimetic **1,** with the *C*‐terminal amide and the *C*_*γ*_ and more distal atoms of Arg and Lys fitted manually, gives an root mean square deviation (RMSD) for the six pairs of *C*_*α*_ and *C*_*β*_ atoms of 0.52 Å (Figure 2e, and see Supporting Information).

**Scheme 1:**
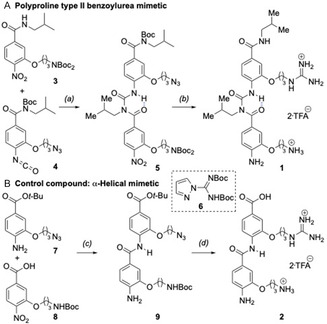
Synthesis of A) PPII benzoylurea mimetic **1**, and B) control compound **2**. *Reagents and conditions:* (a) **3**, KHMDS (1.1 equiv), THF, −78 °C, **4**, 2 h, 86%; (b) conditions X, Y, Z, 87% over three steps; (c) Mukaiyama's reagent (2‐chloro‐1‐methylpyridinium iodide, 1.2 equiv), DIPEA (2.5 equiv), CH_2_Cl_2_, 40 °C, 20 h, 81%; (d) conditions X, Y, Z, 60% over three steps; conditions X: Pd(OH)_2_/C (10 wt%), H_2_, EtOH, r.t.; conditions Y: reagent **6** (*N,N*’–di–Boc‐1*H*‐pyrazole‐1‐carboxamidine, 1.2 equiv), DIPEA (2.5 equiv), CH_2_Cl_2_, 69 h; conditions Z: CH_2_Cl_2_/TFA (1:1 *v*/*v*), 1 h, 99%. HMDS = bis(trimethylsilyl)amide, equiv = equivalent, THF = tetrahydrofuran, DIPEA = *N*,*N*‐diisopropylethylamine, TFA = trifluoroacetic acid.

The synthesis of mimetic **1** was based on a modified version of our previous methods^[^ ^38^ ^]^ using two variously functionalized monomer units. The first, a secondary amide **3**, was deprotonated using KHMDS and the resultant nitrogen‐centered anion was added to an isocyanate **4** to give benzoylurea **5** (**Scheme** **1**A). The use of a strong base necessitated two orthogonal protecting group strategies: i) *N*‐*N*‐diprotection of one of the amine side‐chains with two Boc groups, ii) introduction of the other amine side‐chain in a masked form as an azide. Following benzoylurea formation the azide group was reduced *via* hydrogenation and the resulting amine was transformed into a guanidinium group with *N,N*’‐diBoc‐1*H*‐pyrazole‐1‐carboxamidine **6**, thus providing a mimic of Arg354. Global removal of Boc groups with TFA was followed by purification by *rp*‐HPLC (see Supporting Information) to give PPII helical mimetic **1**.

**Figure 3:**
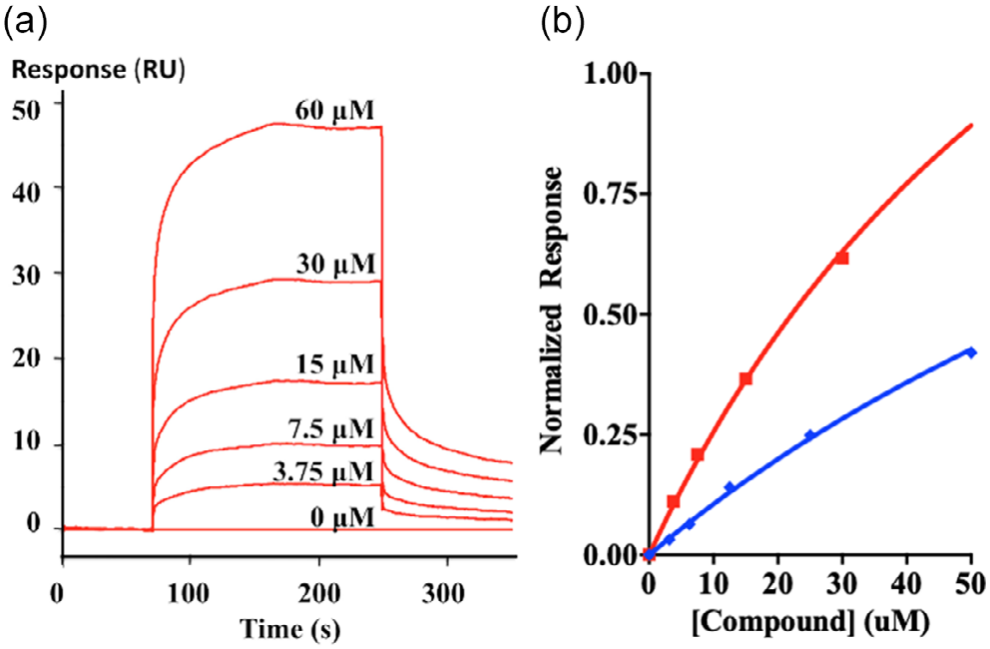
SPR binding results: a) raw sensograms for molecule **1**; b) fits of binding data for mimetic **1** (red) and Gab2a peptide (blue). These indicate K_d_ values of 83 ± 6 and 161 ± 6 μM, respectively.

Binding of **1** to Grb2 was assessed using surface plasmon resonance (SPR). Grb2 protein was covalently bound to a dextran‐coated chip by NHS‐ester coupling, and **1** was flowed over the surface until equilibrium binding was reached (**Figure** **3**, and see Supporting Information). Using a 1:1 binding model the equilibrium binding affinity was 83 ± 6 μM, which compares favorably with the affinity of the Gab2a 12mer (161 ± 6 μM). Aggregation prevented measurement of binding at compound concentrations in excess of 50 μm.

**Figure 4:**
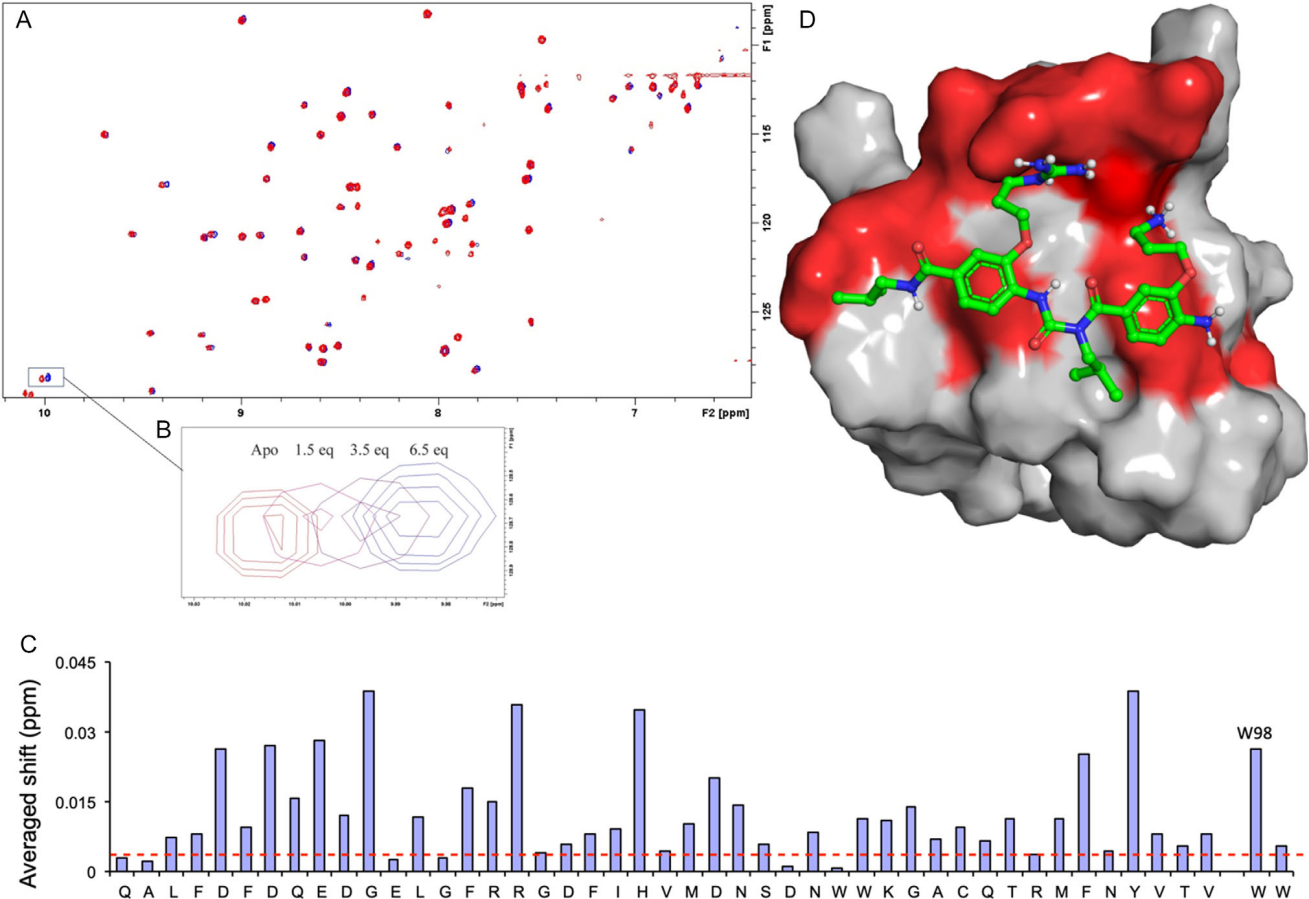
NMR analysis of **1**/Grb2 interaction: A) ^1^H‐^15^N HSQC spectrum of uniformly ^15^N‐labeled Grb2 in the absence (red) and presence (blue) of 6.5 equivalents of mimetic **1**; B) dose‐dependent chemical shift change of the indole ring of residue W98 of Grb2 upon titration of increasing amounts of **1**; C) chemical shift changes for Grb2 with the significance threshold of 0.015 ppm indicated by the red dashed line; and D) mimetic **1** overlayed on the X‐Ray diffraction structure of Grb2 (PDB 2W0Z), with residues found to shift significantly in the HSQC experiment highlighted in red: mimetic **1** has significant interactions with the same region of the Grb2 protein that binds the Gab2a peptide. Bruker AVII 700 MHz (TCI cryoprobe), 298 K, 90:10 H_2_O: D_2_O, 35 mM phosphate buffer pH 7.2 + 45 mM NaCl + 3.0 mM DTT (see Supporting Information).

To better characterize the binding mode and site of the peptidomimetic, we performed nuclear magnetic resonance (NMR) studies of ^15^N Grb2 upon binding of **1** and identified a series of focal, dose‐responsive chemical shift changes that colocalize to a region of Gab2a binding (**Figure** **4**, and see Supporting Information).^[^ ^31^ ^]^ Notably, the absence of other surface chemical shift changes is consistent with minimal promiscuous binding, whilst a lack of perturbations in the hydrophobic core of the protein is consistent with preservation of global folding. To assess if this measured binding is due to nonspecific interactions with the cationic side‐chains of **1**, a control molecule **2** was synthesized displaying the same Arg and Lys side‐chains, based on a benzamide scaffold designed for *α*‐helix mimicry^[^ ^41^ ^]^ that does not match the spatial projection of Gab2a side‐chains (Figure S1, Supporting Information). A similar synthetic approach, based on the coupling of two prefunctionalized aromatic monomers **7** and **8**, was employed to that used for mimetic **1**, except that Mukaiyama's reagent affected fragment union *via* amidation (Scheme 1b and Scheme S4, Supporting Information).^[^ ^42^, ^43^ ^]^ This construct bound extremely weakly to Grb2 by SPR (Figure S2, Supporting Information). ^15^N Grb2 NMR titration experiments with **2** indicated that the most significant shifts are to the core of the protein, including, for example, I21 (Figure S3, Supporting Information), with no large contiguous surface binding sites. Overall, this indicates that control molecule **2**, despite having the same cationic side chains as **1**, fails to bind to Grb2.

In conclusion, we have synthesized the first example of a rationally designed nonpeptidic scaffold mimicking an extended recognition surface of the PPII helix. Decoration of this backbone with hydrophobic and hydrophilic side‐chains provided a peptidomimetic that binds the Grb2 target orthosterically at comparable affinities to the native Gab2a peptide (*K*_d_ 83 μM). Since an α‐helical mimetic displaying the critical Lys and Arg side‐chains on one face did not engage the target, there are promising grounds for improving affinity for Grb2, and other, therapeutically relevant PPII targets while maintaining a high degree of specificity, which will form the basis of our future work.

## Conflict of Interest

The authors declare no conflict of interest.

## Supporting information

Supplementary Material

## Data Availability

The data that support the findings of this study are available in the supplementary material of this article.
