# Supplementary material for: A Polyproline Type II Peptidomimetic Disrupts a Grb2 SH3C Domain Protein–Protein Interaction Implicated in Breast Cancer
Source: Chembiochem. 2025 Jun 6;26(14):e202500343. doi: 10.1002/cbic.202500343 (PMC12278339; doi:10.1002/cbic.202500343)
Supplement: Supplementary file 1 — Supplementary Material [file CBIC-26-e202500343-s001.pdf]

Supporting Information

A Polyproline II Peptidomimetic Disrupts a Grb2 SH3C Domain Protein-protein Interaction Implicated in Breast Cancer

James Luccarelli,[a] Philip C. Simister,[b] Andrew D. Hamilton,[a, e] Stephan M. Feller,[b, c]* and Sam Thompson[a, d]*

[a]*Department of Chemistry, Chemistry Research Laboratory, University of Oxford, Mansfield Road, Oxford, OX1 3TA (UK)*

[b]*Biological Systems Architecture Group, Weatherall Institute of Molecular Medicine, University of Oxford, Oxford OX3 9DS (UK)*

[c] *Institute of Molecular Medicine, Martin-Luther-University Halle-Wittenberg, Halle (Saale) (Germany)*

[d] *School of Chemistry, University of Southampton, Southampton, SO17 1BJ (UK)*

[e] *Department of Chemistry, New York University, 100 Washington Square East, New York, NY 10003 (USA)*

stephan.feller@uk-halle.de, sam.thompson@southampton.ac.uk

CONTENTS

1. Computational Modelling 2

2. General Synthetic Methods 3

3. Synthetic Procedures 4

4. NMR Spectra of Synthetic Compounds 17

4. Protein Expression and Purification 35

5. Surface Plasmon Resonance 36

6. Protein NMR 37

7. Supplemental References 38

1. Computational Modelling

The backbone scaffold conformation on which to model mimic **1** was taken from CCDC #644652. Side-chain mimics of Pro (*i-2*), Arg (*i*), Pro (*i+2*), and Lys (*i+3*) were added manually in Spartan[1] and subjected to a molecular mechanics conformational search to find readily accessible lower energy conformers. One of these low energy conformations was overlayed in PyMol[2] with a truncate of the Gab2a PPII helix extracted from PDB 2W0Z. The RMSD value of 0.52 Å for the Gab2a C_α and C_β positions of the *i*, *i+2*, and *i+3* side-chains and the corresponding positions of mimetic **1** was performed in PyMol using the 'Pair fit' function (main manuscript Figure 2e).

Mimic **2** was constructed in Spartan (Figure S1a)[1] and subjected to a molecular mechanics conformational search to find readily accessible lower energy conformers. One of these low energy conformations was overlayed in PyMol[2] with a truncate of the Gab2a PPII helix extracted from PDB 2W0Z. The RMSD value of 1.26 Å for the Gab2a C_α and C_β positions of the *i* and *i+3* side-chains and the corresponding positions of mimetic **2** was performed in PyMol using the 'Pair fit' function (Figure S1b).

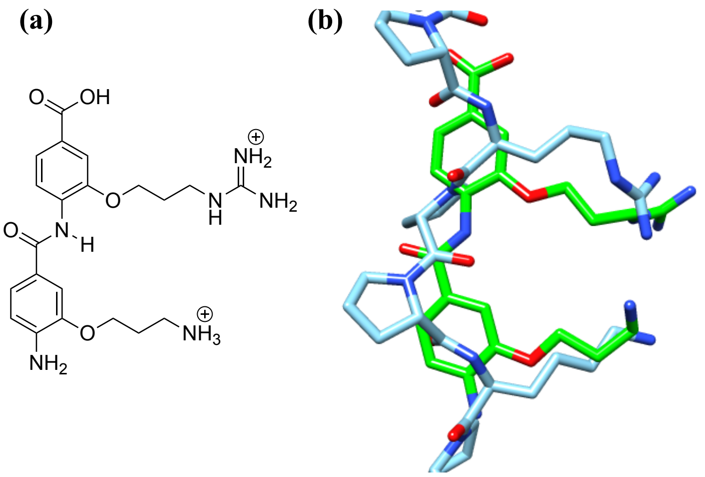

Figure S1: Benzamide mimic **2** designed to reproduce the *i* (arginine) and *i+3* (lysine) side-chains of an α-helix. a) chemical structure. b) superimposition of Gab2a peptide (blue) and mimic **2** (green). The RMSD for the C_α and C_β atoms of the peptide and mimic is 1.26 Å.

2. General Synthetic Methods

Solvents and Reagents

Reactions were carried out under a nitrogen or argon atmosphere in oven-dried glassware unless otherwise stated. Standard inert atmosphere techniques were used in handling all air and moisture sensitive reagents. Anhydrous tetrahydrofuran and dichloromethane (from commercial sources) were dried on an MB-SPS-800 solvent purification system. Other solvents and reagents were used directly as received from commercial suppliers.

Chromatography

Flash column chromatography was carried out using Merck 60 silica gel. Thin-layer chromatography was carried out using Merck Kieselgel 60 F254 (230-400 mesh) fluorescent treated silica, visualized under UV light (254 nm) and by staining with aqueous potassium permanganate solution. High performance liquid chromatography was performed using a 1525 pump, 2707 autosampler, and 2849 detector, all from Waters. Phenomonex Luna columns (250 mm long, 5 μm beads, C18 reverse-phase medium) were used for HPLC separations. Semi-preparative HPLC was run using 10 mL.min^{-1} flow through 21.2 mm-diameter column. Sample injections for semi-preparative runs consisted of 500 μL of solution containing no more than 50 mg of sample. HPLC solvents were degassed by sonication for 30 minutes and contained 0.1 % v/v TFA.

Small Molecule Analysis

^{1}H and ^{13}C NMR spectra were recorded using a Bruker 600, 500, or 400 MHz spectrometer running TopSpin™ software and are quoted in ppm for measurement against residual solvent peaks as internal standards. Chemical shifts (δ) are given in parts per million (ppm), and coupling constants (*J*) are given in Hertz (Hz). The ^{1}H NMR spectra are reported as follows: δ / ppm (multiplicity, coupling constant *J* / Hz (where appropriate), number of protons, assignment). Multiplicity is abbreviated as follows: s = singlet, br = broad, d = doublet, t = triplet, quint. = quintet, m = multiplet. Peaks that could not be assigned in the ^{1}H spectra due to the similarity of aromatic protons are indicated by (Ar-H). Compound names are those generated by ChemBioDraw™ (CambridgeSoft) following IUPAC nomenclature. However, the NMR assignment numbering used is arbitrary and does not follow any particular convention. Numbering of compounds is illustrated on the spectra themselves, *vide infra*. The ^{13}C NMR spectra are reported in δ / ppm and unassigned aromatic peaks are indicated by (Ar) or (Q) for a quaternary aromatic or carbonyl carbon. Two-dimensional (COSY, HSQC, HMBC) NMR spectroscopy was used to assist the assignment of signals in the 1H and 13C NMR spectra. IR spectra were recorded on a Bruker Tensor 27 FT-IR spectrometer from a thin film deposited onto a diamond ATR module. Only selected maximum absorbances (ν_{max}) of the most intense peaks are reported (cm^{-1}). High-resolution mass spectra were recorded on a Bruker MicroTof mass spectrometer (ESI) by the internal service at the Department of Chemistry, University of Oxford. Melting points were recorded using a Leica Galen III hot-stage microscope apparatus and are reported uncorrected in degrees Celsius (˚C).

3. Synthetic Procedures

Scheme S1: Synthetic route to isocyanate fragment **4**.

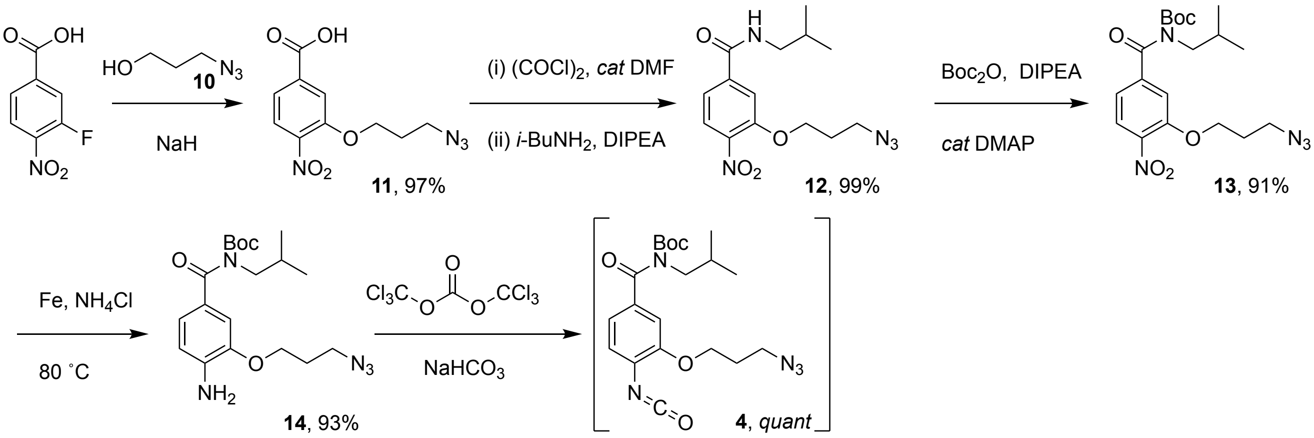

3-Azidopropan-1-ol 10

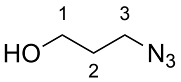

Sodium azide (1.95 g, 30 mmol, 1.05 eq) was dissolved in DMSO (60 mL). To this solution was added 3-bromo-1-propanol (3.97 g, 28.6 mmol, 1.0 eq) and the mixture heated to 70 ˚C and stirred overnight. The reaction was partitioned between water (100 mL) and ether (100 mL) and washed with ether (3 x 60 mL). The organic layers were combined, washed with brine, dried ($MgSO_4$), filtered, and concentrated (500 mBar, 40 ˚C). Purification *via* flash column chromatography (1:1 ether:petrol) gave *the title compound* **10** as a volatile, colourless oil (2.70 g, 26.7 mmol, 93 %): δ_H (500 MHz, $CDCl_3$) 3.82 (2H, q, *J* 5.7, H1), 3.53 (2H, quint, *J* 3.5, H3), 1.90 (2H, quint, *J* 6.3, H2); δ_C (100 MHz, $CDCl_3$) 60.0 (C1), 48.5 (C3), 31.4 (C2); IR υ_{max} 3335, 2946, 2882, 2090, 1045; HRMS (FI) found 101.0590. $C_3H_7N_3O^+$ $[M]^+$ requires 101.0590. Spectroscopic data agreed with literature values.[3]

3-(3-Azidopropoxy)-4-nitrobenzoic acid 11

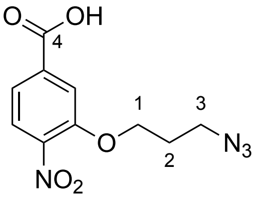

Following the procedure of Boger *et al*,[4] azide **10** (655 mg, 6.5 mmol, 1.2 eq) was added dropwise to a stirred solution of sodium hydride (60 % dispersion in oil, 540 mg, 13.5 mmol, 2.5 eq) in THF (30 mL) at 0 ˚C. The mixture was stirred for 15 minutes at 0 ˚C before 3-fluoro-4-nitrobenzoic acid (1.00 g, 5.4 mmol, 1.0 eq) was added. The reaction was stirred for 5 minutes at 0 ºC, then 2 hours at room temperature, before being diluted with EtOAc (20 mL). NH_4Cl (20 mL) was added, and the mixture was washed with HCl (0.5 M, 20 mL x 3). The organic layer was collected, dried ($MgSO_4$), filtered, and concentrated *in vacuo*. The crude mixture was purified *via* flash column chromatography (3:2 EtOAc:Pet + 1 % AcOH) to give *the title compound* **11** as a yellow solid (1.40 g, 5.26 mmol, 97 %): MP 123-126; δ_H (400 MHz, CD_3OD) 7.86 (1H, d, *J* 10.9, Ar), 7.85 (1H, s, Ar), 7.72 (1H, dd, *J* 8.3; 1.6, Ar), 4.30 (2H, t, *J* 5.9, H1), 3.56 (2H, t, *J* 6.6, H3), 2.09 (2H, quint, *J* 6.2,

H2); δ_C (125 MHz, CD_3OD) 166.6 (C4), 151.6 (Ar), 143.0 (Ar), 136.0 (Ar), 125.1 (Ar), 121.8 (Ar), 115.6 (Ar), 66.6 (C1), 28.5 (C2); IR υ_{max} 3061, 2924, 2853, 2102, 1690, 1589, 1530, 1259; HRMS (ESI) found 265.0569 $C_{10}H_9N_4O_5^-$ $[M-H]^-$ requires 265.0578.

3-(3-Azidopropoxy)-*N*-isobutyl-4-nitrobenzamide 12

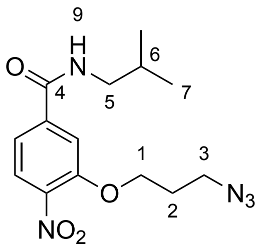

Oxalyl chloride (10 eq) was added to a solution of azido benzoic acid **11** (100 mg, 0.38 mmol, 1.0 eq) dissolved in DCM (5 mL/mmol). DMF (~ 0.5 eq) was added slowly and the mixture stirred vigorously for 1 h. Solvent was removed under reduced pressure, and the resulting oil azeotroped three times with chloroform. The oil was dissolved in DCM (5 mL/mmol) and cooled to 0 ˚C. DIPEA (1.5 eq) was added to the solution dropwise, followed by isobutyl amine (36 mg, 0.49 mol, 1.3 eq) added dropwise. The solution was allowed to warm to room temperature and stirred overnight. Methanol (0.5 mL) was added, and the mixture was washed with NH_4Cl (10 mL/100 mg), and extracted with DCM (3 x 10 mL/100 mg). The organic layers were combined, dried ($MgSO_4$), filtered, and concentrated *in vacuo*. The crude mixture was purified *via* flash column chromatography (2:1 ether:petrol) to give *the title compound* **12** as a viscous orange oil (120 mg, 0.37 mmol, 99 %): δ_H (400 MHz, $CDCl_3$) 7.86 (1H, d, *J* 8.3, Ar), 7.61 (1H, d, *J* 1.6, Ar), 7.28 (1H, dd, *J* 8.1; 1.6, Ar), 6.26 (1H, s, H9), 4.27 (2H, t, *J* 5.8, H1), 3.58 (2H, t, *J* 6.4, H3), 3.30 (2H, t, *J* 6.7, H5), 2.10 (2H, quint, *J* 6.1, H2), 1.92 (1H, sept, *J* 1.9, H6), 0.99 (6H, d, *J* 6.7, H7); δ_C (62.5 MHz, $CDCl_3$) 165.4 (C4), 152.6 (Ar), 141.6 (Ar), 140.6 (Ar), 126.2 (Ar), 118.1 (Ar), 114.5 (Ar), 66.6 (C1), 48.1 (C3), 48.0 (C6), 29.7 (C6), 28.5 (C2), 20.6 (C7); IR υ_{max} 3314, 3083, 2959, 2100, 1645, 1527, 1254; HRMS (ESI) found 344.1320 $C_{14}H_{19}N_5O_4Na^+$ $[M+Na]^+$ requires 344.1329.

***tert*-Butyl (3-(3-azidopropoxy)-4-nitrobenzoyl)(isobutyl)carbamate 13**

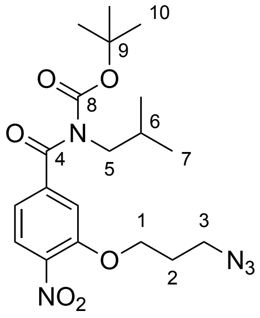

Di-*tert*-butyl dicarbonate (2.0 eq) was added to a solution of amide **12** (625 mg, 1.95 mmol, 1.0 eq) in dichloromethane. DIPEA (3.0 eq) and DMAP (0.1 eq) were added and the reaction stirred at room temperature for 4 days. The mixture was then washed with NH_4Cl and extracted three times with dichloromethane. The organic layers were combined, dried ($MgSO_4$), filtered, and concentrated *in vacuo*. The crude mixture was purified *via* flash column chromatography (1:1 ether:petrol) to give *the title compound* **13** as a yellow oil (751

mg, 1.78 mmol, 91 %): δ_H (250 MHz, $CDCl_3$) 7.88 (1H, d, *J* 8.3, Ar), 7.26 (1H, d, *J* 1.4, Ar), 7.11 (1H, dd, *J* 8.3; 1.1, Ar), 4.24 (2H, t, *J* 5.7, H1), 3.68 (2H, d, *J* 7.4, H5), 3.61 (2H, t, *J* 6.4, H3), 2.22-2.03 (3H, m, H2 and H6), 1.28 (9H, s, H10), 0.99 (6H, d, *J* 6.7, H7); δ_C (62.5 MHz, $CDCl_3$) 171.3 (C4), 153.7 (C8), 152.4 (Ar), 144.0 (Ar), 140.8 (Ar), 125.7 (Ar), 119.1 (Ar), 113.7 (Ar), 84.3 (C9), 66.6 (C1), 53.1 (C5), 48.1 (C3), 28.8 (C6), 28.5 (C2), 27.9 (C10), 20.6 (C7); IR υ_{max} 3332, 2960, 2099, 1735, 1673, 1526, 1146; HRMS (ESI) found 444.1843 $C_{19}H_{27}N_5O_6Na^+$ $[M+Na]^+$ requires 444.1854.

***tert*-Butyl (4-amino-3-(3-azidopropoxy)benzoyl)(isobutyl)carbamate 14**

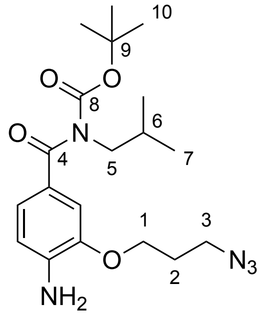

Iron powder (10 eq) and ammonium chloride (0.5 eq) were added to a stirred solution of aromatic nitro compound **13** (551 mg, 1.31 mmol, 1.0 eq) dissolved in ethanol/water (4:1). The mixture was heated to 80 ˚C for 30 minutes, then allowed to cool and filtered through Celite® with EtOAc. Solvent was removed *in vacuo* and the product purified *via* flash column chromatography (2:1 petrol:ether) to yield *the title compound* **14** as a pale tan oil (474 mg, 1.22 mmol, 93 %): δ_H (250 MHz, $CDCl_3$) 7.12 (1H, d, *J* 1.7, Ar), 7.07 (1H, dd, *J* 8.0; 1.8, Ar), 6.63 (1H, d, *J* 8.1, Ar), 4.12 (2H, t, *J* 6.0, H1), 3.59 (2H, d, *J* 7.3, H5), 3.51 (2H, t, *J* 6.6, H3), 2.16-2.00 (3H, m, H2 and H6), 1.24 (9H, s, H10), 0.94 (6H, d, *J* 6.7, H7); δ_C (62.5 MHz, $CDCl_3$) 173.2 (C4), 154.7 (C9), 145.9 (Ar), 141.7 (Ar), 127.0 (Ar), 123.0 (Ar), 113.0 (Ar), 111.6 (Ar), 82.1 (C9), 65.2 (C1), 53.1 (C5), 48.4 (C3), 28.7 (C6), 28.5 (C2), 27.8 (C10), 20.3 (C7); IR υ_{max} 3487, 3372, 2962, 2099, 1721, 1633, 1617, 1141; HRMS (ESI) found 414.2101 $C_{19}H_{29}N_5O_4Na^+$ $[M+Na]^+$ requires 414.2112.

***tert*-Butyl (3-(3-azidopropoxy)-4-isocyanatobenzoyl)(isobutyl)carbamate 4**

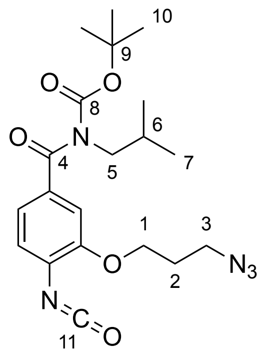

Sodium bicarbonate (2.0 eq) and bis(trichloromethyl) carbonate (0.6 eq) were added to a vigorously stirred solution of a substituted aniline **14** (85 mg, 0.22 mmol) in 1:1 DCM:water (2 mL /100 mg). The mixture was stirred for 30 minutes, then diluted with NH_4Cl (10 mL/100 mg) and DCM (10 mL/100 mg). The mixture was extracted with DCM (3 x 10 mL/100mg) and the organic layers combined, dried (Na_2SO_4), filtered, and solvent removed *in vacuo* to afford *the title compound* **4** as a yellow oil (102 mg, 0.22 mmol, *quant*), which was

immediately used for subsequent reaction without further purification: δ$_H$ (250 MHz, $CDCl_3$) 7.26 (1H, d, *J* 1.5, Ar), 7.19 (1H, dd, *J* 8.0; 1.6, Ar), 7.13 (1H, d, *J* 8.1, Ar), 4.32 (2H, t, *J* 5.8, H1), 3.76 (2H, d, *J* 7.4, H5), 3.71 (2H, t, *J* 6.4, H3), 2.33-2.14 (3H, m, H2 and H6), 1.37 (9H, s, H10), 1.09 (6H, d, *J* 6.7, H7); δ$_C$ (62.5 MHz, $CDCl_3$) 172.7 (C4), 154.4 (C8), 152.8 (Ar), 136.2 (Ar), 131.4 (Ar), 126.7 (C11), 123.3 (Ar), 121.0 (Ar), 111.0 (Ar), 83.4 (C9), 66.0 (C1), 53.3 (C5), 48.2 (C3), 28.9 (C6), 28.7 (C2), 27.9 (C10), 20.6 (C7); IR υ_{max} 2962, 2242, 2098, 1731, 1670, 1332, 1144.

Scheme S2: Synthetic route to secondary amide fragment **3**.

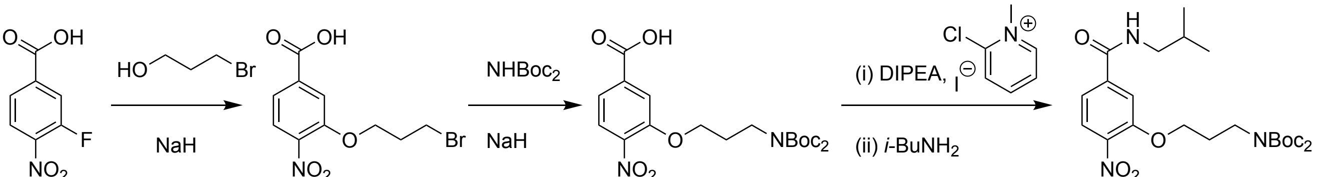

3-(3-Bromopropoxy)-4-nitrobenzoic acid 15

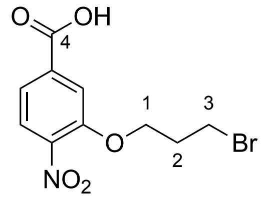

Adapting the procedure of Boger *et al*,[4] 3-fluoro-4-nitrobenzoic acid (1.00 g, 5.4 mmol, 1.0 eq) was dissolved in THF (40 mL) and cooled to 0 ˚C. Sodium hydride (670 mg, 16.7 mmol, 3.1 eq) was added in portions and the mixture stirred for 5 minutes before 3-bromo-1-propanol (1.5 g, 10.8 mmol, 2.0 eq) was added over 3 h by syringe pump. The reaction was then warmed to room temperature and stirred for 16 h, before being diluted with EtOAc (100 mL). HCl (1M, 60 mL) was added, and the mixture was washed with HCl (0.5 M, 20 mL x 3). The organic layer was collected, dried ($MgSO_4$), filtered, and concentrated, and the residue purified *via* flash column chromatography (3:2 Pet:EtOAc + 1 % AcOH) to afford *the title compound* **15** as a pale yellow solid (1.44 g, 4.7 mmol, 87 %): MP 124-127; δ$_H$ (400 MHz, CD_3OD) 7.87-7.82 (2H, m, Ar), 7.72 (1H, dd, *J* 8.3; 1.6, Ar), 4.32 (2H, t, *J* 5.7, H1), 3.66 (2H, t, *J* 6.4, H3), 2.35 (2H, quint, *J* 6.1, H2); δ$_C$ (100 MHz, CD_3OD) 166.5 (C4), 151.6 (Ar), 136.0 (Ar), 132.3 (Ar), 125.0 (Ar), 121.8 (Ar), 115.7 (Ar), 67.4 (C1), 32.2 (C2), 29.2 (C3); IR υ_{max} 2960, 2886, 2605, 1726, 1688, 1525, 1304, 1251, 743; HRMS (ESI) found 301.9677, 303.9659 $C_{10}H_9BrNO_5^-$ $[M-H]^-$ requires 301.9670 (^{79}Br), 303.9650 (^{81}Br).

3-(3-(Bis(*tert*-butoxycarbonyl)amino)propoxy-4-nitrobenzoic acid 16

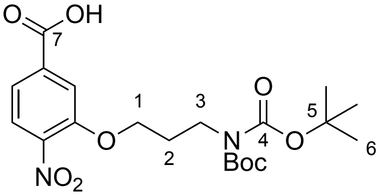

Di-*tert*-butyl iminodicarboxylate (633 mg, 2.92 mmol, 1.1 eq) was added to a solution of sodium hydride (72 mg, 3 mmol, 1.1 eq) in DMF (10 mL) and the mixture heated to 65 ˚C. Alkyl bromide **15** was dissolved in THF (10 mL) and sodium hydride (72 mg, 3 mmol as a 60 % dispersion in oil, 1.1 eq) added. This solution was stirred for 5 minutes then added *via* syringe to the heated solution containing the di-*tert*-butyl iminodicarboxylate. The mixture was stirred for 16 h at 65 ˚C before being allowed to cool to room temperature. The mixture was diluted with EtOAc, washed with HCl (0.2 M, 10 mL x 3), and the organic layer dried (Na_2SO_4), filtered, concentrated, and purified *via* flash column chromatography (3:2 EtOAc:petrol + 1 % AcOH) to yield *the title compound* **16** as a viscous yellow oil (1.08 g, 2.45 mmol, 92 %): δ_H (400 MHz, CD_3OD) 7.87-7.80 (2H, m, Ar), 7.71 (1H, t, *J* 7.7, Ar), 4.25 (2H, t, *J* 5.8, H1), 3.81 (2H, t, *J* 6.9, H3), 2.11 (2H, quint, *J* 6.2, H2), 1.48 (18H, s, H6); δ_C (100 MHz, CD_3OD) 166.6 (C7), 153.0 (Ar), 151.7 (C4), 143.0 (Ar), 135.8 (Ar), 125.0 (Ar), 121.7 (Ar), 115.5 (Ar), 82.9 (C5), 67.8 (C1), 43.9 (C3), 28.7 (C2), 27.3 (C6); IR υ_{max} 3300, 2980, 1785, 1704, 1493, 1367, 1254, 1099; HRMS (ESI) found 439.1703 $C_{20}H_{27}N_2O_9^-$ $[M-H]^-$ requires 439.1722.

3-(3-(Bis(*tert*-butoxycarbonyl)amino)propoxy- *N*-isobutyl-4-nitrobenzamide 3

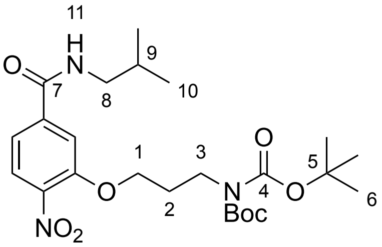

DIPEA (2.5 eq) was added dropwise to a solution of carboxylic acid **16** (960 mg, 2.18 mmol, 1.0 eq) in DCM. Mukaiyama's reagent (2-chloro-1-methylpyridinium iodide, 1.2 eq) was added to the mixture and the reaction heated to 40 ˚C for 30 minutes. Isobutyl amine (207 mg, 2.83 mmol, 1.3 eq) was then added and the reaction stirred for 20 hours at 40 ˚C. The solution was diluted with NH_4Cl (10 mL/100 mg) and DCM (10 mL/100 mg) and extracted with DCM (3 x 10 mL/100 mg). The organic layers were combined, dried (sodium sulfate), filtered, and purified *via* flash column chromatography (3:2 petrol:ether) to yield *the title compound* **3** as a pale oil (729 mg, 1.47 mmol, 67 %): δ_H (400 MHz, $CDCl_3$) 7.82 (1H, d, *J* 8.3, Ar), 7.56 (1H, d, *J* 1.4, Ar), 7.29 (1H, dd, *J* 8.3; 1.5, Ar), 6.44 (1H, t, *J* 5.3, H11), 4.20 (2H, t, *J* 6.1, H1), 3.79 (2H, t, *J* 7.1, H3), 3.28 (2H, t, *J* 6.7, H8), 2.11 (2H, quint, *J* 6.3, H2), 1.91 (1H, sept, *J* 6.7, H9), 1.47 (18H, s, H6), 0.97 (6H, d, *J* 6.7, H10); δ_C (100 MHz, $CDCl_3$) 165.5 (C7), 152.5 (C4), 152.3 (Ar), 141.3 (Ar), 140.0 (Ar), 125.6 (Ar), 117.8 (Ar), 114.0 (Ar), 82.5 (C5), 67.8 (C1), 47.6 (C8), 43.6 (C3), 28.6 (C2), 28.5 (C9), 28.0 (C6), 20.1 (C10); IR υ_{max} 3337, 3084, 2963, 1772, 1645, 1528, 1366, 1110. HRMS (ESI) found 518.2473 $C_{24}H_{37}N_3O_8Na^+$ $[M+Na]^+$ requires 518.2473.

Scheme S3: Synthetic route to PPII helical peptidomimetic **1**.

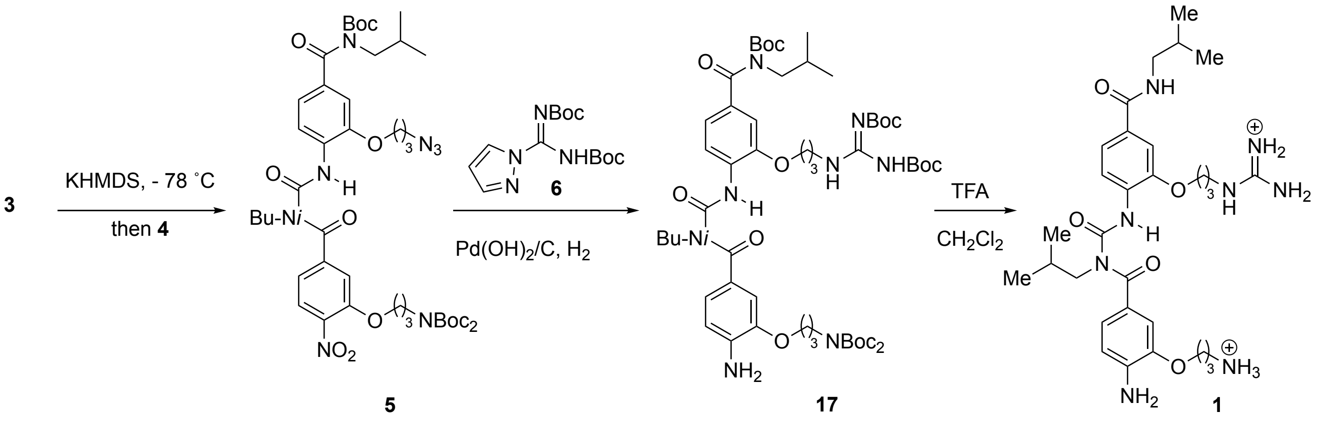

***tert*-Butyl(3-(3-azidopropoxy)-4-(3-(3-(3-(bis(*tert*-butoxycarbonyl)amino)propoxy)-4-nitrobenzoyl)-3-isobutylureido)benzoyl)(isobutyl)carbamate 5**

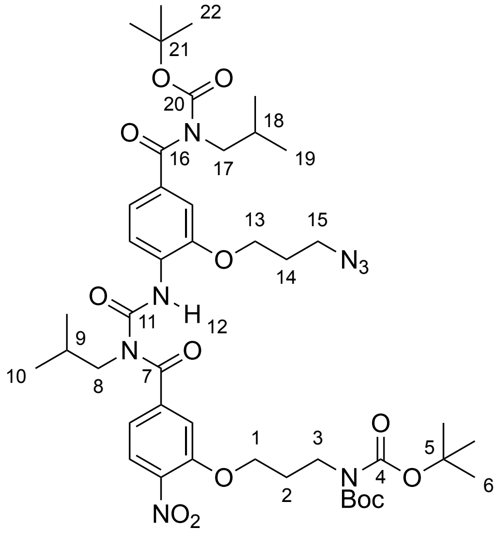

Potassium hexamethyldisilazide (KHMDS, 0.5 M in toluene, 1.1 eq) was added dropwise to a stirred solution of amide **3** (521 mg, 1.05 mmol, 1.0 eq) in THF (2 mL/mmol) at -78 ˚C. A solution of isocyanate **4** (429 mg, 1.03 mmol) in THF (2 mL/mmol) was added dropwise and the mixture stirred for 2 h at -78 ˚C. AcOH (0.1 mL) was added dropwise, and the solution was allowed to warm to room temperature. The solution was diluted with NH_4Cl (10 mL/100 mg) and DCM (10 mL/100 mg) and extracted with DCM (3 x 10 mL/100 mg). The organic layers were combined, dried (sodium sulfate), filtered, and purified *via* flash column chromatography (1:1 petrol:ether) to yield *the title compound* **5** as a viscous orange oil (835 mg, 0.89 mmol, 86 %): δ_H (500 MHz, $CDCl_3$) 11.61 (1H, s, H12), 8.37 (1H, d, *J* 8.3, Ar), 7.91 (1H, d, *J* 8.3, Ar), 7.21-7.14 (3H, m, Ar), 7.11 (1H, dd, *J* 8.2; 1.4, Ar), 4.18 (4H, t, *J* 5.8, H1 and H13), 3.82 (2H, t, *J* 6.9, H3), 3.69-3.62 (6H, m, H17, H8, H15), 2.20-2.06 (5H, m, H2, H14, and H9 or H18), 1.98 (1H, sept, *J* 6.8, H9 or H18), 1.49 (18H, s, H6 and H22), 0.97 (6H, d, *J* 6.8, H10 or H19), 0.82 (6H, d, *J* 6.7, H10 or H19); δ_C (125 MHz, $CDCl_3$) 173.2 (C16 or C7), 172.7 (C16 or C7), 154.2 (C11), 152.6 (C4 or C20), 152.5 (C4 or C20), 152.4 (Ar), 151.1 (Ar), 147.3 (Ar), 141.1 (Ar), 140.7 (Ar), 132.8 (Ar), 130.4 (Ar), 125.9 (Ar), 121.2 (Ar), 118.6 (Ar), 113.3 (Ar), 110.4 (Ar), 82.7 (C21), 82.5 (C5), 68.1 (C1 or C13), 65.4 (C1 or C13), 54.1 (C8 or C17), 53.0 (C8 or C17), 48.0 (C15), 43.5 (C3), 28.7 (C9 or C18), 28.6 (C2 and C14), 28.3 (C9 or C18), 28.0 (C6), 27.5 (C22), 20.2 (C10 or C19), 19.9 (C10 or C19);

IR υ_{max} 3223, 2964, 2099, 1716, 1531, 1367, 1333, 1148, 1039; HRMS (ESI) found 935.4472 $C_{44}H_{64}N_8O_{13}Na^+$ $[M+Na]^+$ requires 935.4485.

***tert*-Butyl(4-(3-(4-amino-3-(3-(bis(*tert*-butoxycarbonyl)amino)propoxy)benzoyl)-3-isobutylureido)-3-(3-((2,2,10,10-tetramethyl-4,8-dioxo-3,9-dioxa-5,7-diazaundecan-6-yl)amino)propoxy)benzoyl)(isobutyl)carbamate 17**

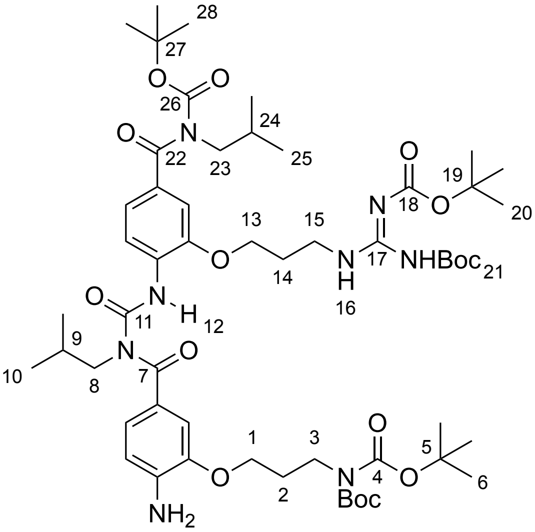

N,N'-diBoc-1*H*-pyrazole-1-carboxamidine **6** (171 mg, 0.55 mmol, 1.2 eq) and palladium on activated carbon (210 mg, 5 % Pd by mass) were added to a stirred solution of benzoylurea **5** (420 mg, 0.46 mmol, 1.0 eq) in EtOAc (20 mL). The solution was flushed three times with hydrogen gas then allowed to stir under an atmosphere of hydrogen for 69 hours. The solution was filtered through Celite® with EtOAc (200 mL), concentrated, and purified *via* flash column chromatography (1:1 ether:petrol) to give *the title compound* **17** as a white foam (442 mg, 0.40 mmol, 87 %): δ_H (500 MHz, $CDCl_3$) 11.48 (1H, s, H12 or H21), 11.43 (1H, s, H12 or H21), 8.46 (1H, t, *J* 5.2, H16), 8.37 (1H, d, *J* 8.8, Ar), 7.17-7.13 (2H, m, Ar), 7.05 (1H, dd, *J* 8.2; 1.6, Ar), 6.94 (1H, d, *J* 1.6, Ar), 6.66 (1H, d, *J* 8.2, Ar), 4.31 (2H, br, NH_2), 4.11 (2H, t, *J* 6.2, H13), 4.07 (2H, t, *J* 5.8, H1), 3.83 (2H, t, *J* 6.8, H3), 3.78 (2H, d, *J* 7.2, H8), 3.70 (2H, qt, *J* 6.4, H15), 3.62 (2H, d, *J* 7.4, H23), 2.19 (2H, quint, *J* 6.5, H14), 2.15-2.06 (3H, m, H2 and H24), 1.97 (1H, sept, *J* 6.8, H9), 1.49 (9H, s, H6, or H20, or H28), 1.48 (18H, s, H6, or H20, or H28), 1.46 (9H, s, H6, or H20, or H28), 1.21 (9H, s, H6, or H20, or H28), 0.95 (6H, d, *J* 6.8, H25), 0.78 (6H, d, *J* 6.7, H10); δ_C (125 MHz, $CDCl_3$) 175.5 (C22), 173.0 (C7), 163.5 (C17), 156.3 (C18), 154.3 (C11), 153.1 (C26), 152.6 (C4), 147.3 (Ar), 145.5 (Ar), 140.3 (Ar), 135.7 (Ar), 132.1 (Ar), 131.3 (Ar), 122.7 (Ar), 121.3 (Ar), 118.1 (Ar), 113.3 (Ar), 111.1 (Ar), 110.5 (Ar), 83.0, 82.6, 82.4 (C5, C19, C27), 66.3 (C13), 66.2 (C1), 55.1 (C8), 53.0 (C23), 43.8 (C3), 37.6 (C15), 29.3 (C2), 28.8 (C14), 28.7 (C24), 28.5 (C9), 28.3, 28.3, 28.0, 27.5 (C6, C20, C28), 20.2 (C25), 19.9 (C10); IR υ_{max} 3366, 2963, 1724, 1639, 1619, 1368, 1330, 1133; HRMS (ESI) found 1099.6287 $C_{55}H_{87}N_8O_{15}^+$ $[M+H]^+$ requires 1099.6285.

3-(2-Amino-5-(((2-(3-((amino(iminio)methyl)amino)propoxy)-4-(isobutylcarbamoyl)phenyl) carbamoyl)(isobutyl)carbamoyl)phenoxy)propan-1-aminium•2 TFA 1

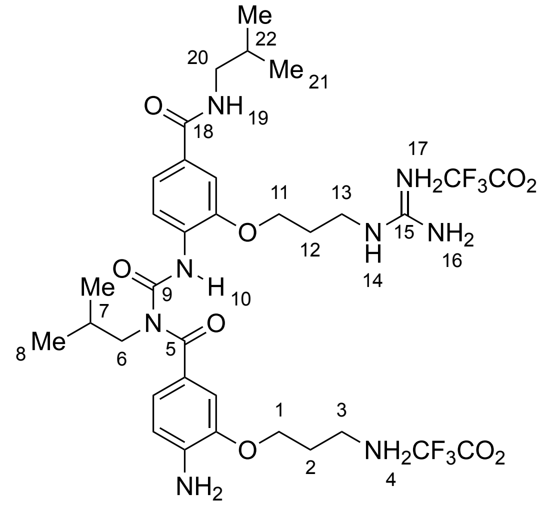

TFA (1.0 mL) was added to a stirred solution of *N*-Boc protected benzoylurea **17** (79 mg, 0.072 mmol) in DCM (1 mL). The mixture was stirred at room temperature for 1 h and toluene (5 mL) was added. The solvent was removed *in vacuo* and the resulting compound azeotroped three times with chloroform. to yield a brown oil (59 mg, 0.071 mmol, 99 %). A small amount was purified *via* semi-preparative HPLC (35/65 CH_3CN/H_2O to 95/5 CH_3CN/H_2O over 40 minutes) to give *the title compound* **1** (18 mg, 0.03 mmol) as a pale yellow oil: δ_H (500 MHz, CD_3OD) 8.32 (1H, d, *J* 8.4, Ar), 7.52 (1H, d, *J* 1.5, Ar), 7.50 (1H, dd, *J* 8.2; 1.8, Ar), 7.13 (1H, dd, *J* 8.0; 1.4, Ar), 7.10 (1H, d, *J* 1.5, Ar), 6.86 (1H, d, *J* 8.1, Ar), 4.24 (2H, t, *J* 5.4, H11), 4.19 (2H, t, *J* 5.5, H1), 3.85 (2H, d, *J* 7.2, H6), 3.58 (2H, t, *J* 7.1, H13), 3.26-3.19 (4H, m, H3 and H20), 2.26-2.15 (4H, m, H2 and H12), 1.92-1.91 (2H, m, H7 and H22), 0.99 (6H, d, *J* 6.7, H21), 0.83 (6H, d, *J* 6.7, H8); δ_C (125 MHz, CD_3OD) 176.6 (C5), 168.6 (C18), 161.0 (q, *J* 36, F_3CCO_2), 157.7 (C15), 153.0 (C9), 148.0 (Ar), 145.8 (Ar), 141.2 (Ar), 131.0 (Ar), 129.9 (Ar), 124.0 (Ar), 123.3 (Ar), 120.2 (Ar), 119.0 (Ar), 114.1 (Ar), 111.4 (Ar), 110.3 (Ar), 65.8 (C1 and C11), 55.2 (C17), 47.5 (C11), 38.5 (C13), 37.5 (C3), 28.8 (C12), 28.7 (C7 and C22), 27.4 (C2), 19.6 (C21), 19.2 (C8); IR υ_{max} 3360, 3191, 2962, 1673, 1631, 1527, 1193, 1137; HRMS (ESI) found 599.3668 $C_{30}H_{47}N_8O_5^+$ $[M+H]^+$ requires 599.3664.

Scheme S4: Synthetic route to α-helical mimic **2** (control compound).

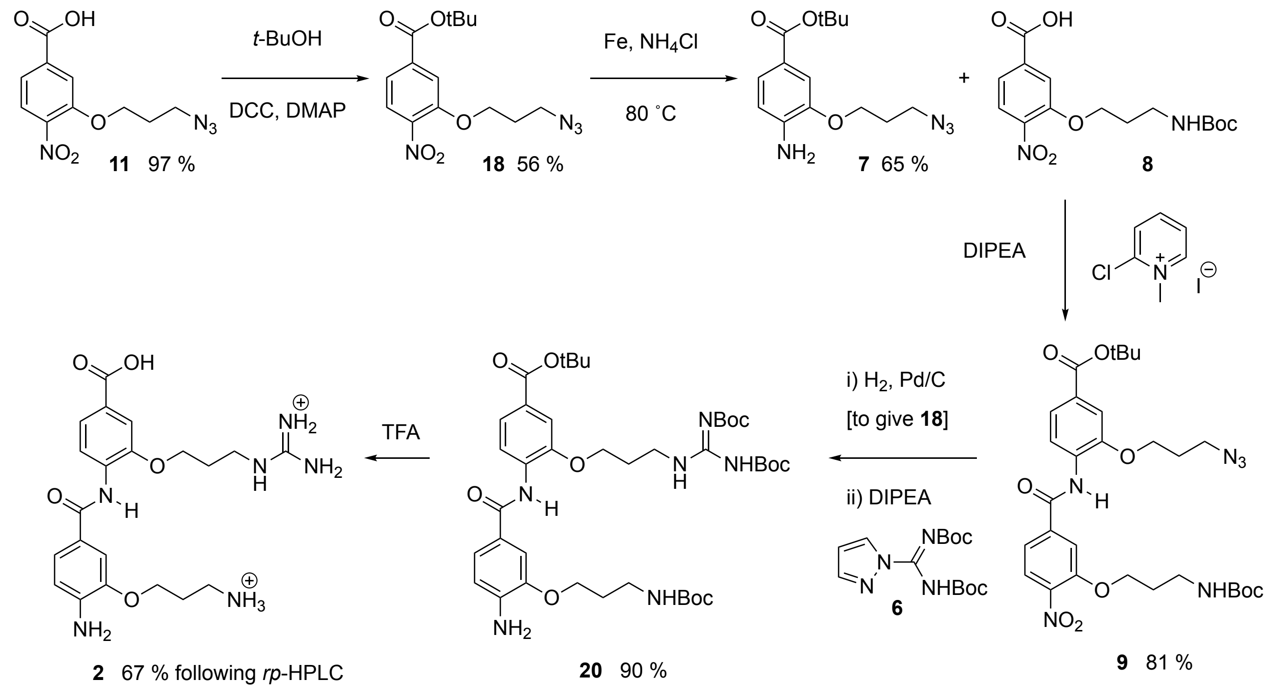

tert-Butyl 3-(3-azidopropoxy)-4-nitrobenzoate 18

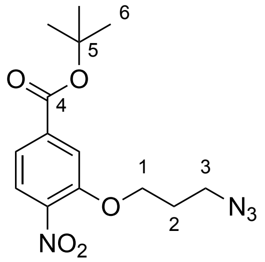

Carboxylic acid **11** (1.4 g, 5.26 mmol, 1.0 eq) was dissolved in DCM (30 mL) and cooled to 0 ˚C and DMAP (96 mg, 0.79 mmol, 0.15 eq), *tert*-butanol (2 mL, 21 mmol, 4.0 eq), and *N,N'*-dicyclohexylcarbodiimide (1.25 g, 6.00 mmol, 1.15 eq) were added. The mixture was warmed to room temperature and stirred for 68 hours before 10 % NaOH (100 mL) and DCM (100 mL) were added. The organic layer was washed with water (2 x 100 mL) and brine (1 x 100 mL), then dried ($MgSO_4$) and purified *via* flash column chromatography (10:1 petrol:ether) to yield *the title compound* **18** as a yellow solid (952 mg, 2.95 mmol, 56 %): MP 74-75; δ_H (400 MHz, $CDCl_3$) 7.78 (1H, d, *J* 8.36, Ar), 7.67 (1H, d, *J* 1.3, Ar), 7.60 (1H, dd, *J* 8.3; 1.5, Ar), 4.23 (2H, t, *J* 5.8, H1), 3.65 (1H, t, *J* 6.5, H3), 2.07 (2H, quint, *J* 6.1, H2), 1.58 (9H, s, H 6); δ_C (100 MHz, $CDCl_3$) 163.6 (C4), 151.5 (Ar), 142.0 (Ar), 136.9 (Ar), 125.2 (Ar), 121.4 (Ar), 115.3 (Ar), 82.5 (C5), 66.1 (C1), 47.7 (C3), 28.4 (C2), 28.0 (C6); IR υ_{max} 3113, 3079, 2986, 2086, 1719, 1611, 1521, 1249; HRMS (ESI) found 345.1163 $C_{14}H_{18}N_4O_5Na^+$ $[M+Na]^+$ requires 345.1169.

***tert*-Butyl 4-amino-3-(3-azidopropoxy)benzoate 7**

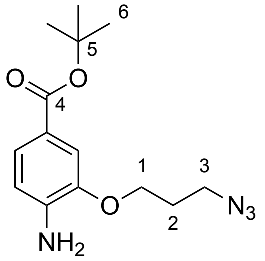

Iron powder (1.65 g, 29.5 mmol, 10 eq) and ammonium chloride (79 mg, 1.48 mmol, 0.5 eq) were added to a stirred solution of the aromatic nitro compound **18** (952 mg, 2.95 mmol) dissolved in ethanol/water (40 mL:10 mL). The mixture was heated to 80 ºC for 30 minutes then filtered through Celite® with EtOAc (150 mL). Solvent was removed *in vacuo* and the product purified *via* flash column chromatography (3:2 petrol:ether) to yield the title compound **7** as a colourless oil (560 mg, 1.92 mmol, 65 %): δ_H (250 MHz, $CDCl_3$) 7.48 (1H, dd, *J* 8.1; 1.7, Ar), 7.40 (1H, d, *J* 1.7, Ar), 6.62 (1H, d, *J* 8.2, Ar), 4.24 (2H, br s, NH_2), 4.08 (2H, t, *J* 6.0, H1), 3.46 (2H, t, *J* 6.6, H3), 2.04 (2H, quint, *J* 6.3, H2), 1.55 (9H, s, H6); δ_C (62.5 MHz, $CDCl_3$) 166.6 (C4), 145.4 (Ar), 141.4 (Ar), 124.5 (Ar), 121.7 (Ar), 113.7 (Ar), 112.6 (Ar), 80.6 (C5), 65.3 (C1), 48.8 (C3), 29.0 (C2), 28.7 (C6); IR υ_{max} 3488, 3369, 2976, 2932, 2098, 1691, 1617, 1297; HRMS (ESI) found 315.1425 $C_{14}H_{20}N_4O_3Na^+$ $[M+Na]^+$ requires 315.1428.

***tert*-Butyl 3-(3-azidopropoxy)-4-(3-(3-((*tert*-butoxycarbonyl)amino)propoxy)-4-nitrobenzamido) benzoate 9**

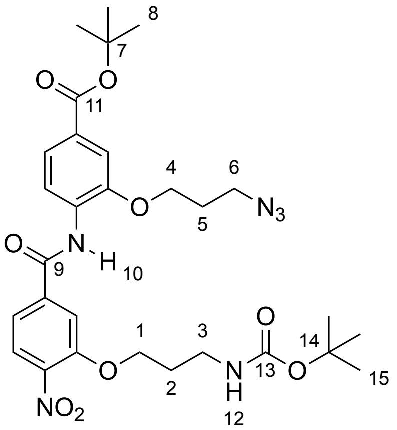

DIPEA (2.5 eq) was added dropwise to a solution of 3-(3-((*tert*-butoxycarbonyl)amino)propoxy)-4-nitrobenzoic acid **8** (443 mg, 1.30 mmol, 1.0 eq) in DCM. Mukaiyama's reagent (2-chloro-1-methylpyridinium iodide, 1.2 eq) was added to the mixture and the reaction heated to 40 ˚C for 30 minutes. Aniline **7** (400 mg, 1.37 mmol, 1.05 eq) was then added and the reaction stirred for 20 h at 40 ˚C. The solution was diluted with NH_4Cl (10 mL.100 mg^{-1}) and DCM (10 mL.100 mg^{-1}) and extracted with DCM (3 x 10 mL.100 mg^{-1}). The organic layers were combined, dried (sodium sulfate), filtered, and purified *via* flash column chromatography (2:1 ether:petrol) to give *the title compound* **9** (651 mg, 1.06 mmol, 81 %) as a white solid: MP 147-148; δ_H (300 MHz, $CDCl_3$) 8.75 (1H, s, H10), 8.47 (1H, d, *J* 8.5, Ar), 7.87 (1H, d, *J* 8.3, Ar), 7.67-7.60 (2H, m, Ar), 7.51 (1H, s, Ar), 7.38 (1H, d, *J* 8.3, Ar), 5.08 (1H, s, H12), 4.26-4.18 (4H, m, H1 and H4), 3.51 (2H, t, *J* 6.1, H6), 3.31 (2H, q, *J* 5.8, H3), 2.12 (2H, quint, *J* 6.1, H5), 2.02 (2H, quint, *J* 5.8, H2), 1.56 (9H, s, H8), 1.38 (9H,

s, H15); δ_C (75 MHz, $CDCl_3$) 165.2, 163.2 (C9, C11), 156.1 (C13), 152.4 (Ar), 146.8 (Ar), 141.4 (Ar), 140.0 (Ar), 131.0 (Ar), 127.9 (Ar), 126.0 (Ar), 123.3 (Ar), 119.0 (Ar), 117.7 (Ar), 114.2 (Ar), 111.5 (Ar), 81.3 (C7), 79.1 (C14), 68.0, 66.6 (C1, C4), 48.9 (C6), 37.8 (C3), 29.0 (C2), 28.5 (C15), 28.4 (C8); IR υ_{max} 3434, 3338, 2978, 2101, 1711, 1681, 1519, 1162; HRMS (ESI) found 637.2603 $C_{29}H_{38}N_6O_9Na^+$ $[M+Na]^+$ requires 637.2592.

***tert*-Butyl 4-(4-amino-3-(3-((*tert*-butoxycarbonyl)amino)propoxy)benzamido)-3-(3-aminopropoxy)benzoate 19**

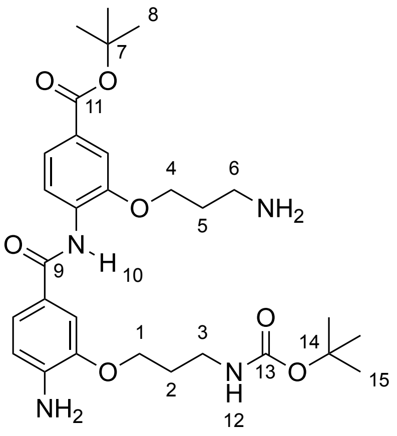

Palladium on activated carbon (10 % by mass; 10 % loading) was added to a stirred solution of benzamide **9** (651 mg, 1.06 mmol) in EtOH. The solution was flushed three times with hydrogen gas then allowed to stir under an atmosphere of hydrogen overnight. The solution was filtered through Celite® with DCM (50 $mL.mmol^{-1}$) and the solvent removed *in vacuo* to yield *the title compound* **19** as a grey foam (632 mg, 1.13 mmol, *quant*), which was used for subsequent reactions without further purification: δ_H (300 MHz, $CDCl_3$) 8.63 (1H, s, H10), 8.50 (1H, d, *J* 8.5, Ar), 7.58 (1H, dd, *J* 8.5; 1.7, Ar), 7.47 (1H, d, *J* 1.7, Ar), 7.32 (1H, d, *J* 1.7, Ar), 7.22 (1H, dd, *J* 7.5, 1.7, Ar), 6.64 (1H, d, *J* 8.1, Ar), 5.10 (2H, s, $ArNH_2$), 4.23 (2H, s, NH_2), 4.14 (2H, t, *J* 6.1, H1 or H4), 4.06 (2H, t, *J* 6.0, H1 or H4), 3.26 (2H, q, *J* 6.1, H3), 2.88 (2H, t, *J* 6.9, H6), 2.00-1.90 (4H, m, H2 and H5), 1.52 (9H, s, H8), 1.36 (9H, s, H15); δ_C (75 MHz, $CDCl_3$) 165.6, 165.1 (C9, C11), 156.1 (C13), 146.7 (Ar), 145.8 (Ar), 140.7 (Ar), 132.3 (Ar), 126.4 (Ar), 124.0 (Ar), 123.3 (Ar), 120.4 (Ar), 118.2 (Ar), 113.5 (Ar), 111.5 (Ar), 110.6 (Ar), 80.9, 79.2 (C7, C14), 66.8, 66.0 (C1, C4), 39.2 (C6), 37.6 (C3), 33.0, 29.6 (C2, C5), 28.4 (C15), 28.2 (C8); IR υ_{max} 3360, 2976, 1697, 1515, 1255, 1165; HRMS (ESI) found 559.3127 $C_{29}H_{43}N_4O_7^+$ $[M+H]^+$ requires 559.3126.

***tert*-Butyl 4-(4-amino-3-(3-((*tert*-butoxycarbonyl)amino)propoxy)benzamido)-3-(3-(2,3-bis(*tert*-butoxycarbonyl)guanidino)propoxy)benzoate 20**

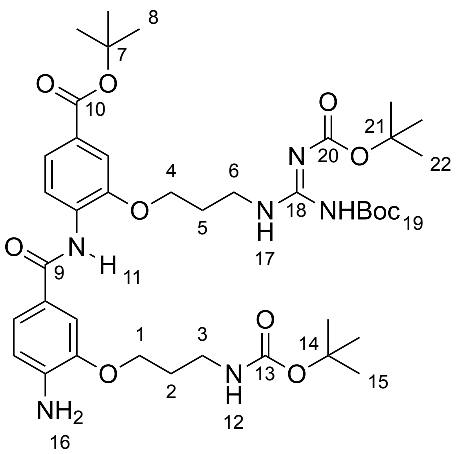

DIPEA (2.5 eq) and *N,N'*-di-Boc-1*H*-pyrazole-1-carboxamidine **6** (1.2 eq) were added to a solution of primary amine **19** (592 mg, 1.06 mmol, 1.0 eq) in DCM. The reaction was stirred for 5 days before water (10 mL/100 mg) and DCM (10 mL/100 mg) were added. The mixture was extracted with DCM (2 x 10 mL/100 mg), dried ($MgSO_4$), filtered, and purified *via* flash column chromatography (3:1 ether:petrol) to yield *the title compound* **20** (767 mg, 0.96 mmol, 90 %) as a white foam: δ_H (500 MHz, $CDCl_3$) 11.46 (1H, s, H19), 8.65 (1H, s, H11), 8.52-8.47 (2H, m, Ar and H17), 7.62 (1H, d, *J* 7.8, Ar), 7.49 (1H, s, Ar), 7.43 (1H, s, Ar), 7.28 (1H, dd, *J* 8.2; 0.9, Ar), 6.68 (1H, d, *J* 8.2, Ar), 4.92 (1H, s, H12), 4.29 (2H, s, H16), 4.16 (2H, t, *J* 5.8, H4), 4.09 (2H, t, *J* 5.9, H1), 3.66 (2H, q, *J* 6.1, H6), 3.30 (2H, q, *J* 5.8, H3), 2.12 (2H, quint, *J* 6.1, H5), 1.97 (2H, quint, *J* 6.1, H2), 1.56 (9H, s, H8), 1.39 (27H, s, H22 and H15); δ_C (125 MHz, $CDCl_3$) 166.0, 165.5 (C9, C10), 163.9 (C18), 156.8, 156.5, 153.7 (C13, C20), 147.2 (Ar), 146.2 (Ar), 141.1 (Ar), 132.6 (Ar), 127.0 (Ar), 124.3 (Ar), 123.8 (Ar), 120.9 (Ar), 119.3 (Ar), 113.9 (Ar), 111.9 (Ar), 111.3 (Ar), 83.8 (C14 or C21), 81.3 (C7), 79.8, 79.6 (C14 or C21), 66.7 (C4), 66.4 (C1), 39.4 (C6), 38.2 (C3), 30.0 (C2), 29.4 (C5), 28.8, 28.6, 28.4 (C15, C22, C8); IR υ_{max} 3342, 2977, 1710, 1617, 1515, 1273, 1165; HRMS (ESI) found 823.4224 $C_{40}H_{60}N_6O_{11}Na^+$ $[M+Na]^+$ requires 823.4212.

4-(4-Amino-3-(3-aminopropoxy)benzamido)-3-(3-guanidinopropoxy)benzoic acid•2TFA 2

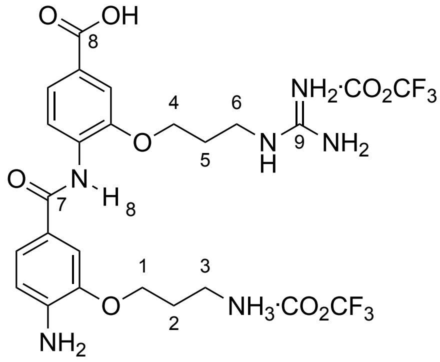

TFA (1.0 mL) was added to a stirred solution of *N*-Boc protected benzamide **20** (60 mg, 0.075 mmol) in DCM (1 mL). The mixture was stirred at room temperature for 80 minutes and toluene (5 mL) was added. The solvent was removed *in vacuo* and the resulting compound azeotroped three times with chloroform. A portion of the residue was purified *via* semi-preparative HPLC (15 % MeCN to 45 % MeCN over 40 minutes) to give

the title compound **2** (10 mg, 0.015 mmol, 67%) as a yellow oil: δ_H (500 MHz, D_2O) 7.80 (1H, d, *J* 8.3, Ar), 7.56 (1H, d, *J* 8.2, Ar), 7.48 (1H, d, *J* 6.7, Ar), 7.46-7.37 (3H, m, Ar), 4.25 (2H, t, *J* 5.6, H1 or H4), 4.14-4.08 (2H, m, H1 or H4), 3.30 (2H, t, *J* 6.7, H3 or H6), 3.26 (2H, t, *J* 7.5, H3 or H6), 2.24 (2H, quint, *J* 7.8, H2 or H5), 2.04 (2H, quint, *J* 6.3, H2 or H5); δ_C (125 MHz, D_2O) 169.4 (C7 or C8), 166.9 (C7 or C8), 162.8 (q, *J* 35.6, $CF_3\mathit{C}O_2$), 156.7 (C9), 151.0 (Ar), 149.7 (Ar), 134.1 (Ar), 130.3 (Ar), 127.3 (Ar), 124.4 (Ar), 123.3 (Ar), 122.9 (Ar), 122.8 (Ar), 120.2 (Ar), 116.2 (q, *J* 291.7, $\mathit{C}F_3CO_2$), 113.0 (Ar), 111.6 (Ar), 66.0 (C1 or C4), 65.9 (C1 or C4), 38.1 (C3 or C6), 36.9 (C3 or C6), 27.6 (C2 or C5), 26.4 (C2 or C5); IR υ_{max} 3361, 3172, 2959, 1674, 1603, 1198, 1136; HRMS (ESI) found 445.2193 $C_{21}H_{29}N_6O_5^+$ $[M+H]^+$ requires 445.2194.

4. NMR Spectra of Synthetic Compounds

3-Azidopropan-1-ol 10

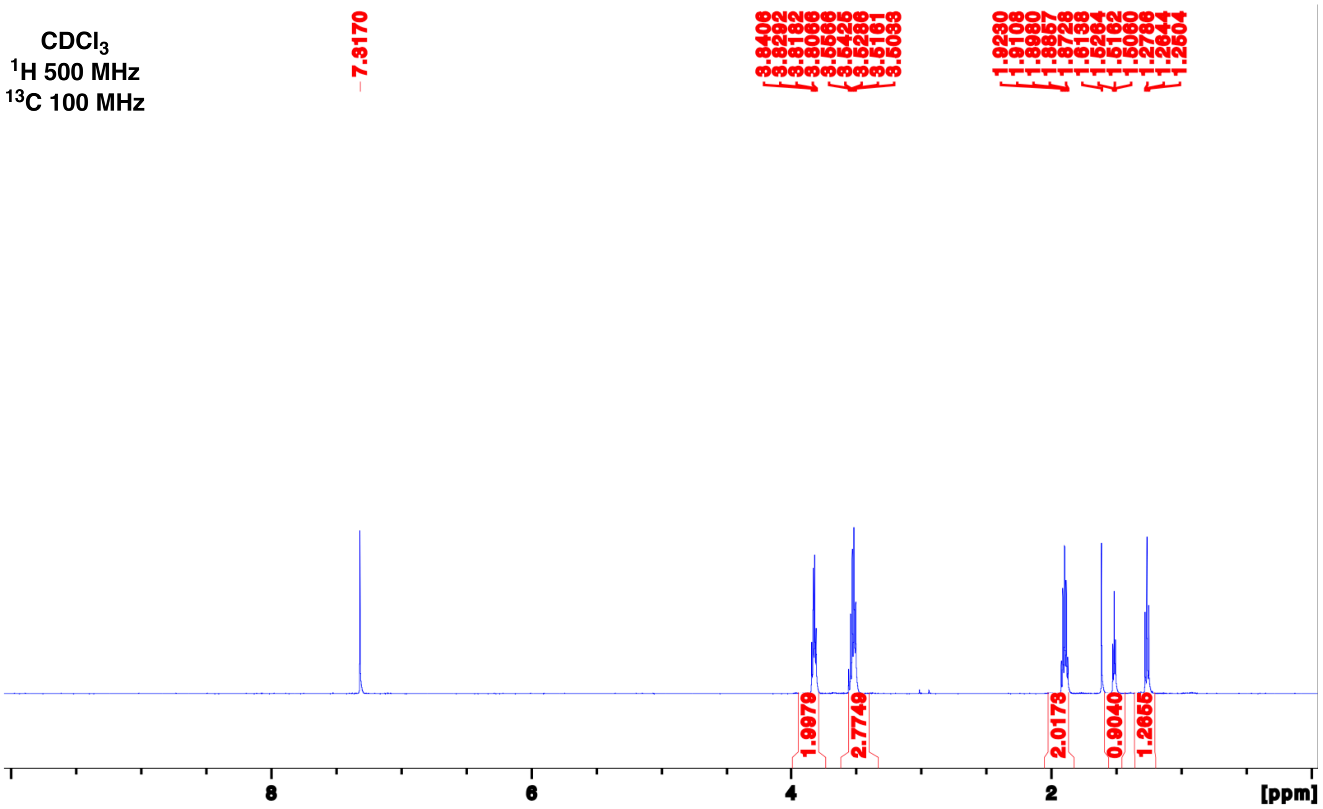

3-(3-Azidopropoxy)-4-nitrobenzoic acid 11

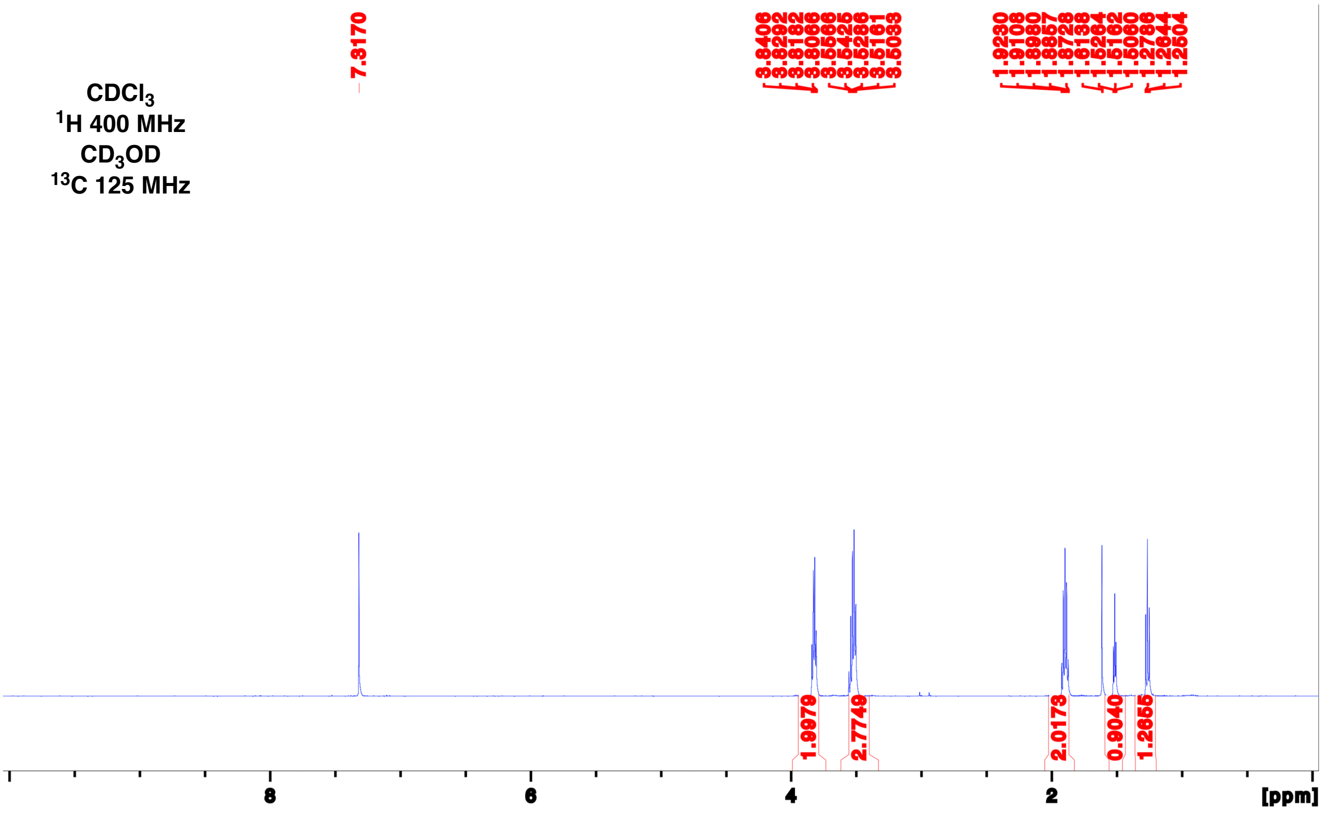

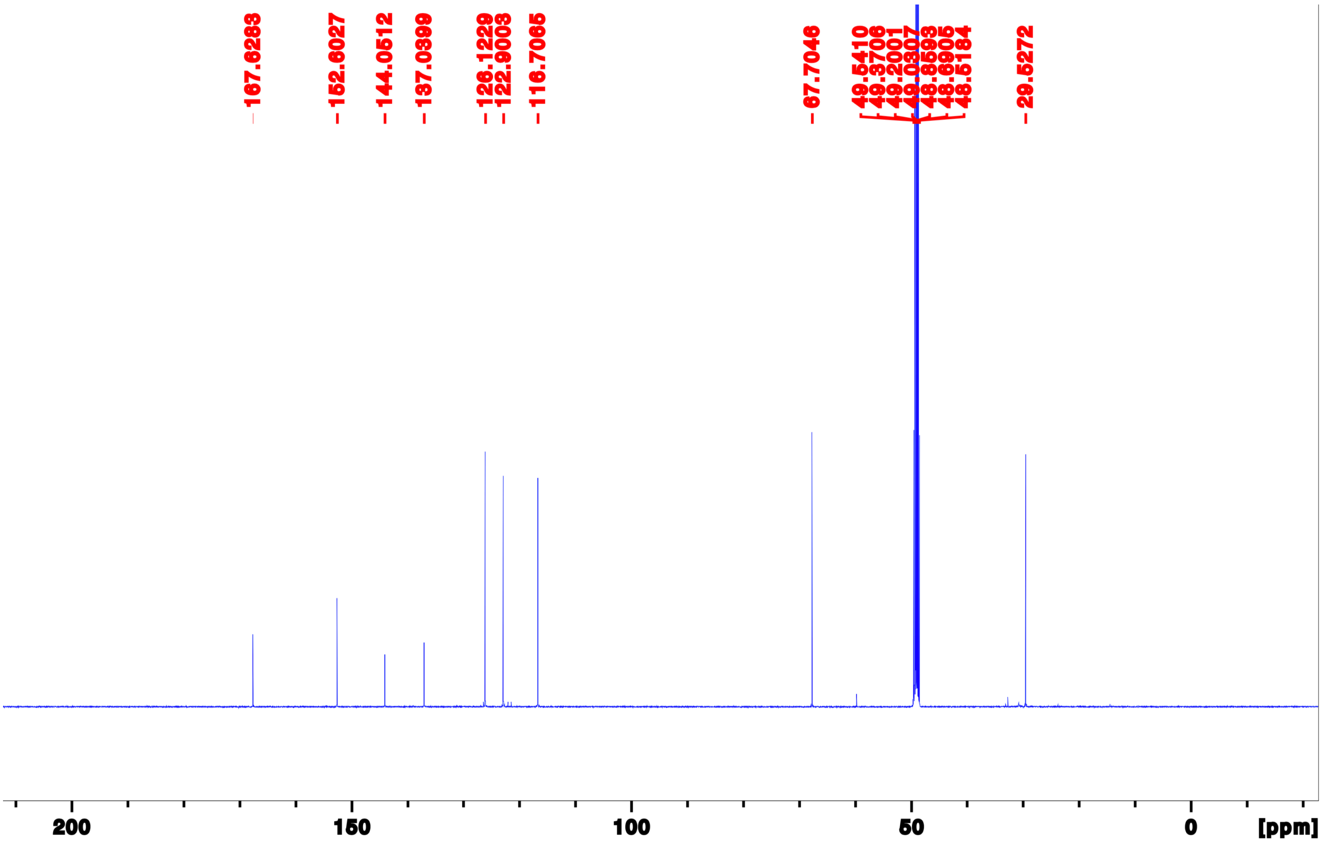

3-(3-Azidopropoxy)-*N*-isobutyl-4-nitrobenzamide 12

CDCl$_3$
^{1}H 400 MHz
^{13}C 100 MHz

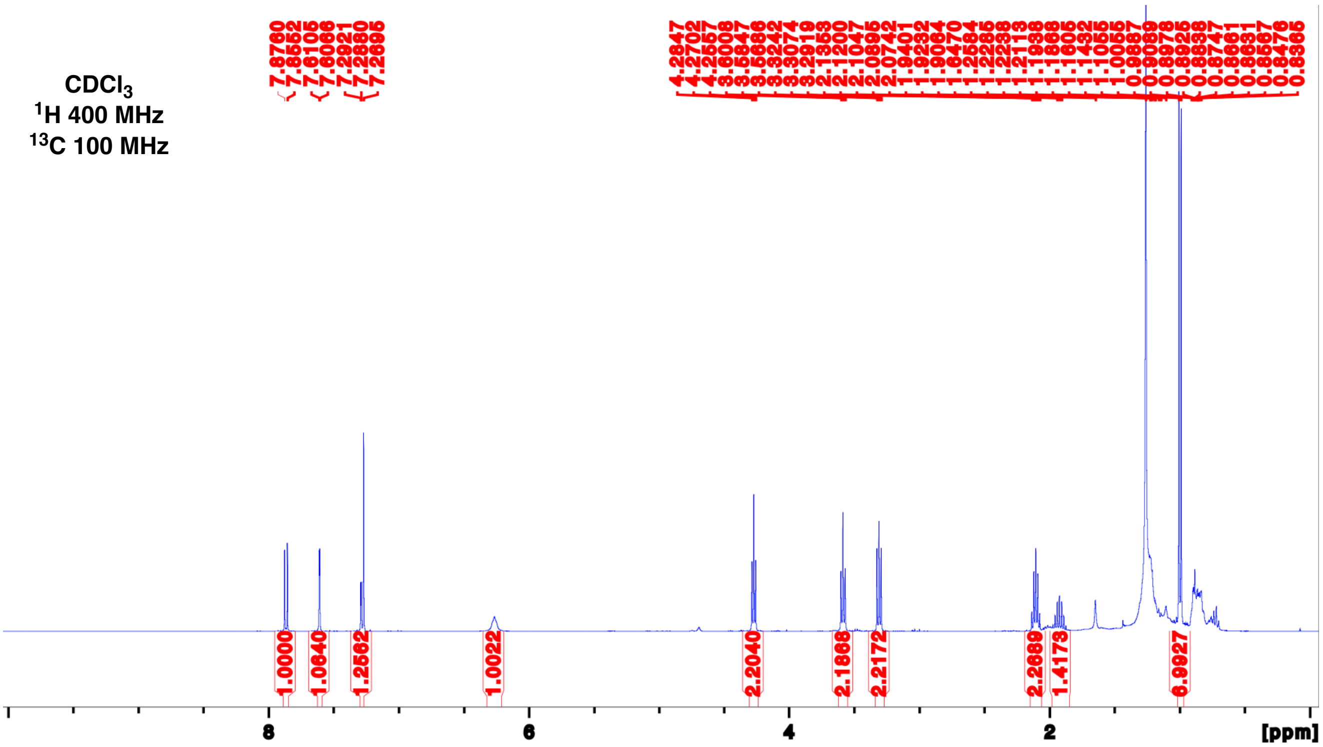

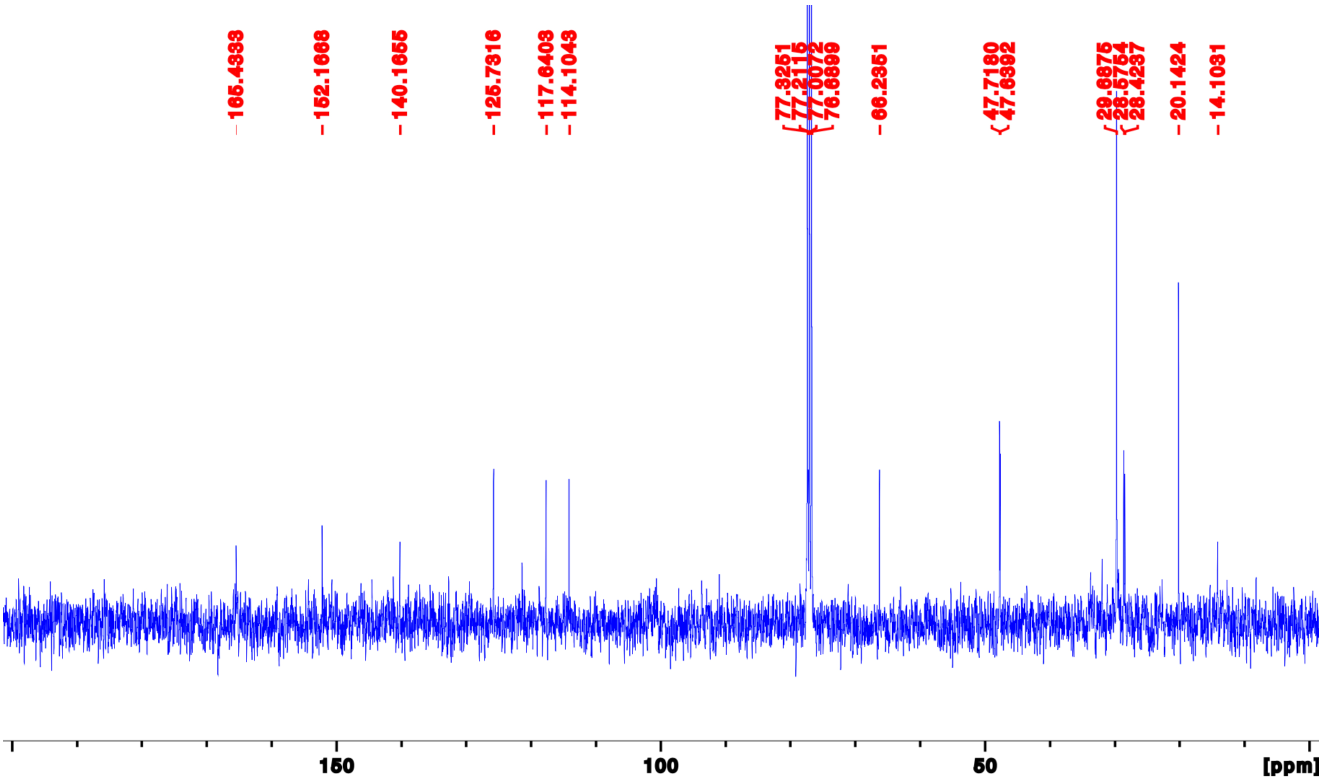

***tert*-Butyl (3-(3-azidopropoxy)-4-nitrobenzoyl)(isobutyl)carbamate 13**

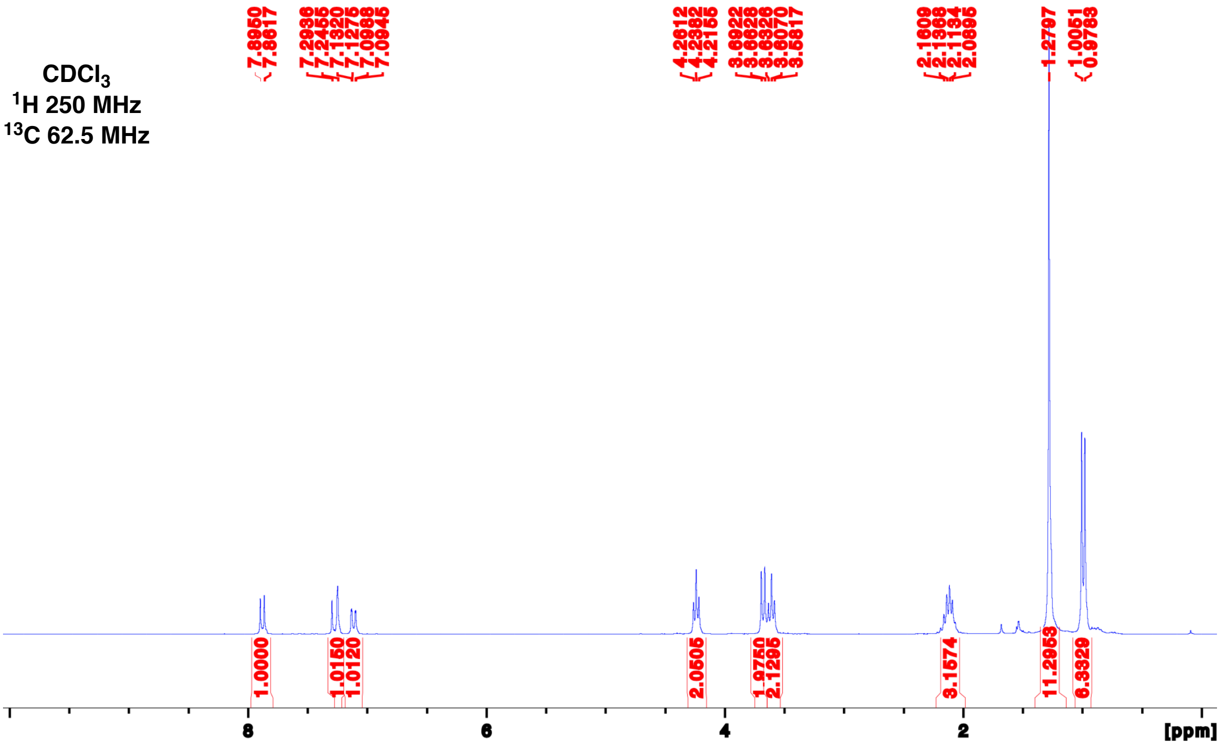

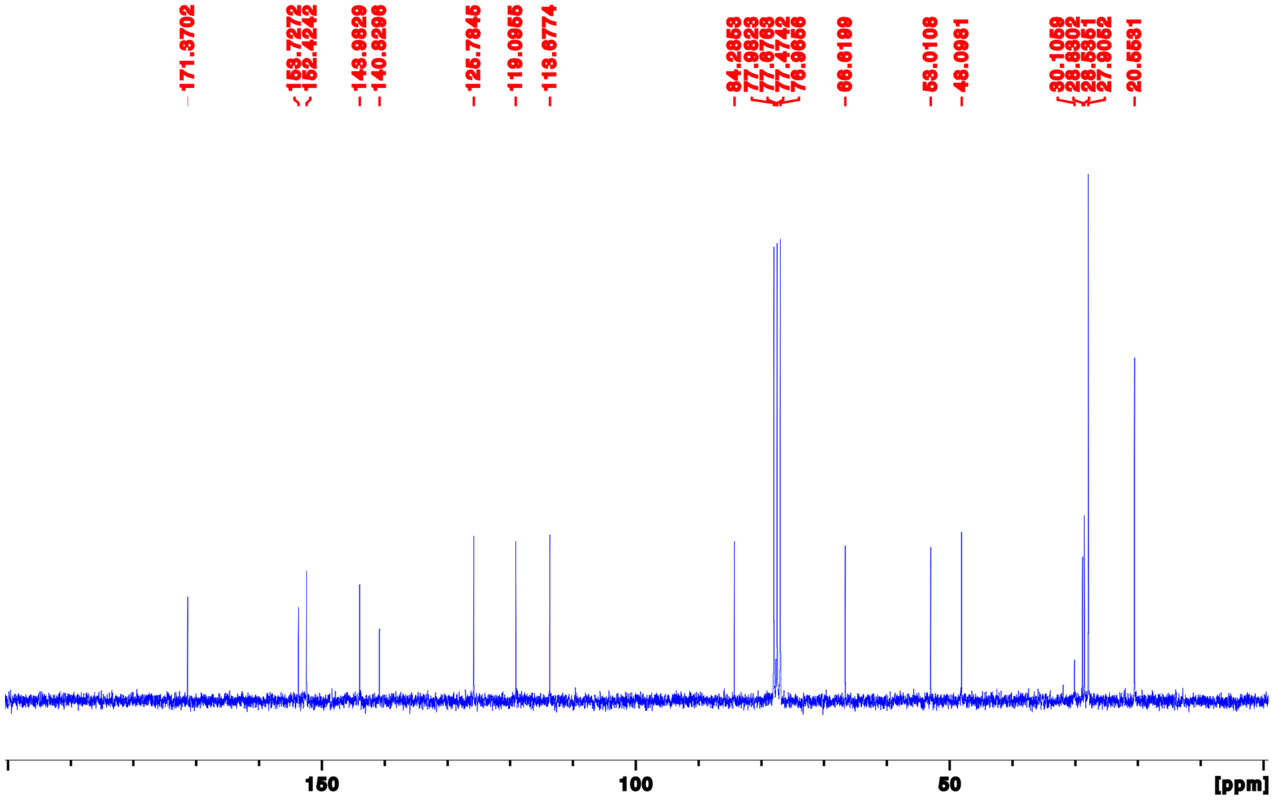

tert-Butyl (4-amino-3-(3-azidopropoxy)benzoyl)(isobutyl)carbamate 14

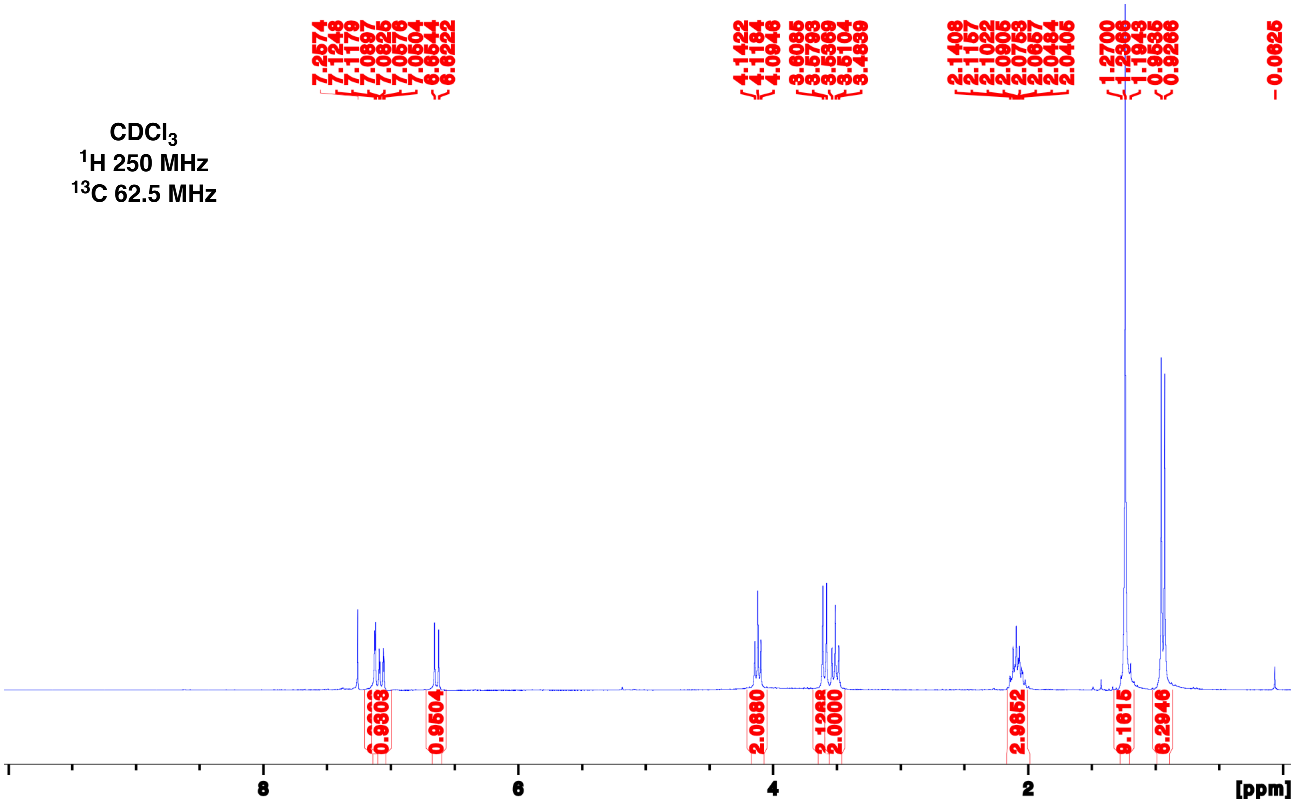

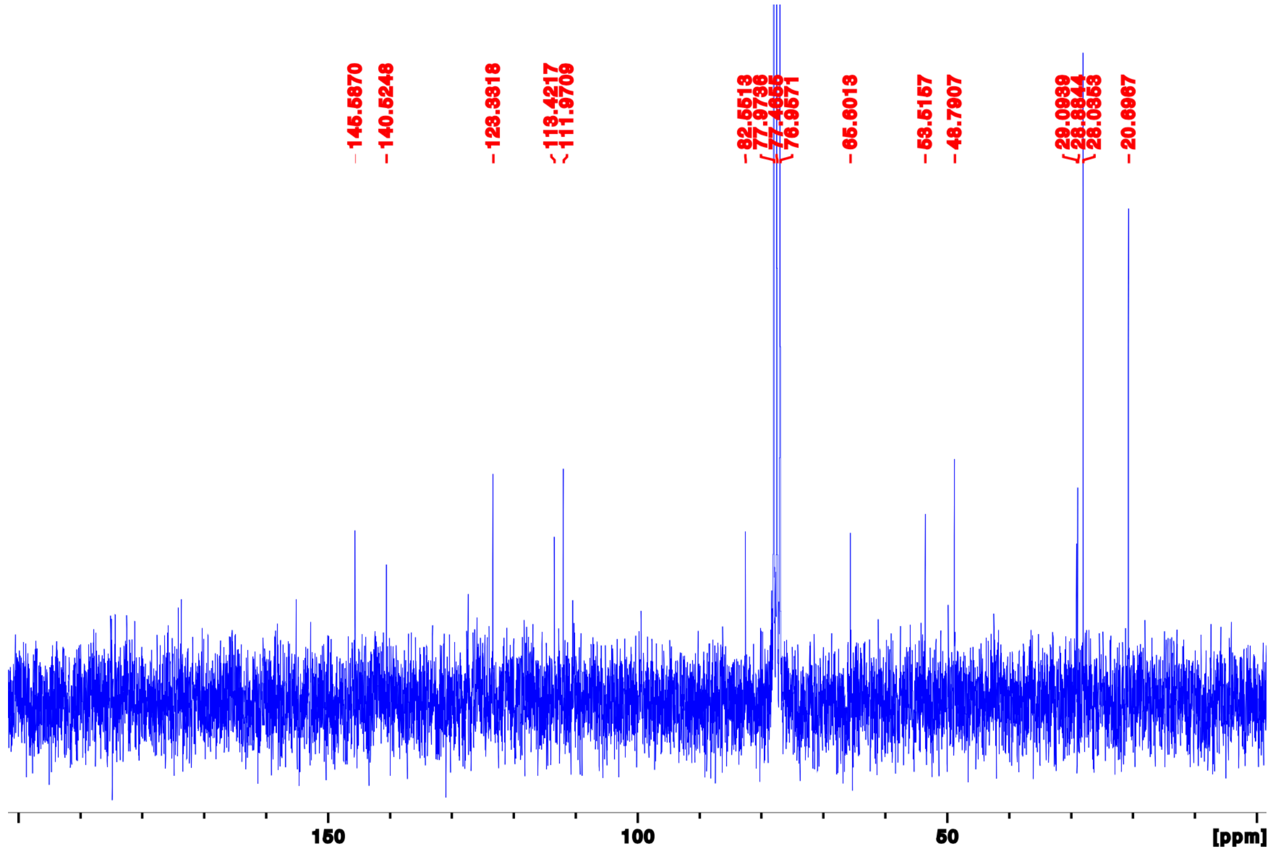

tert-Butyl (3-(3-azidopropoxy)-4-isocyanatobenzoyl)(isobutyl)carbamate 4

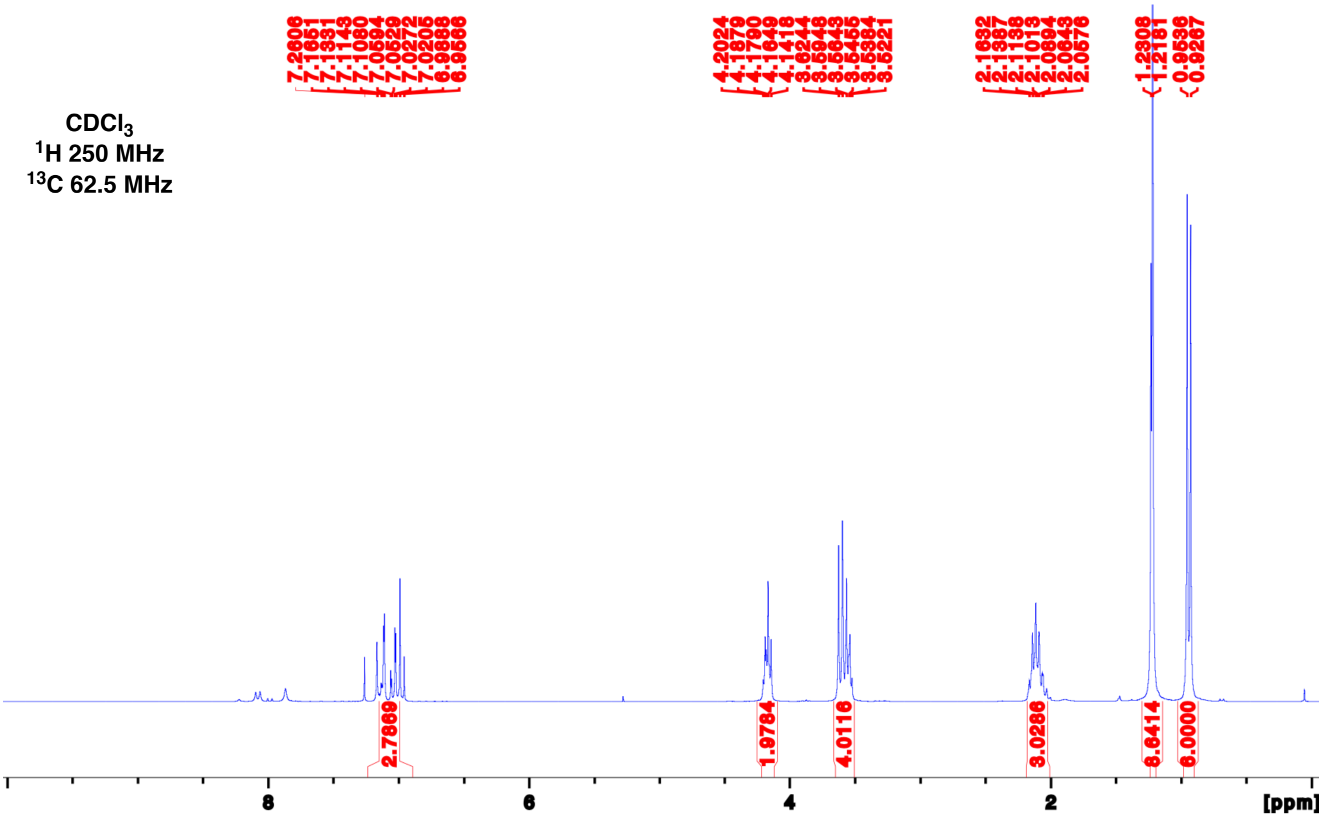

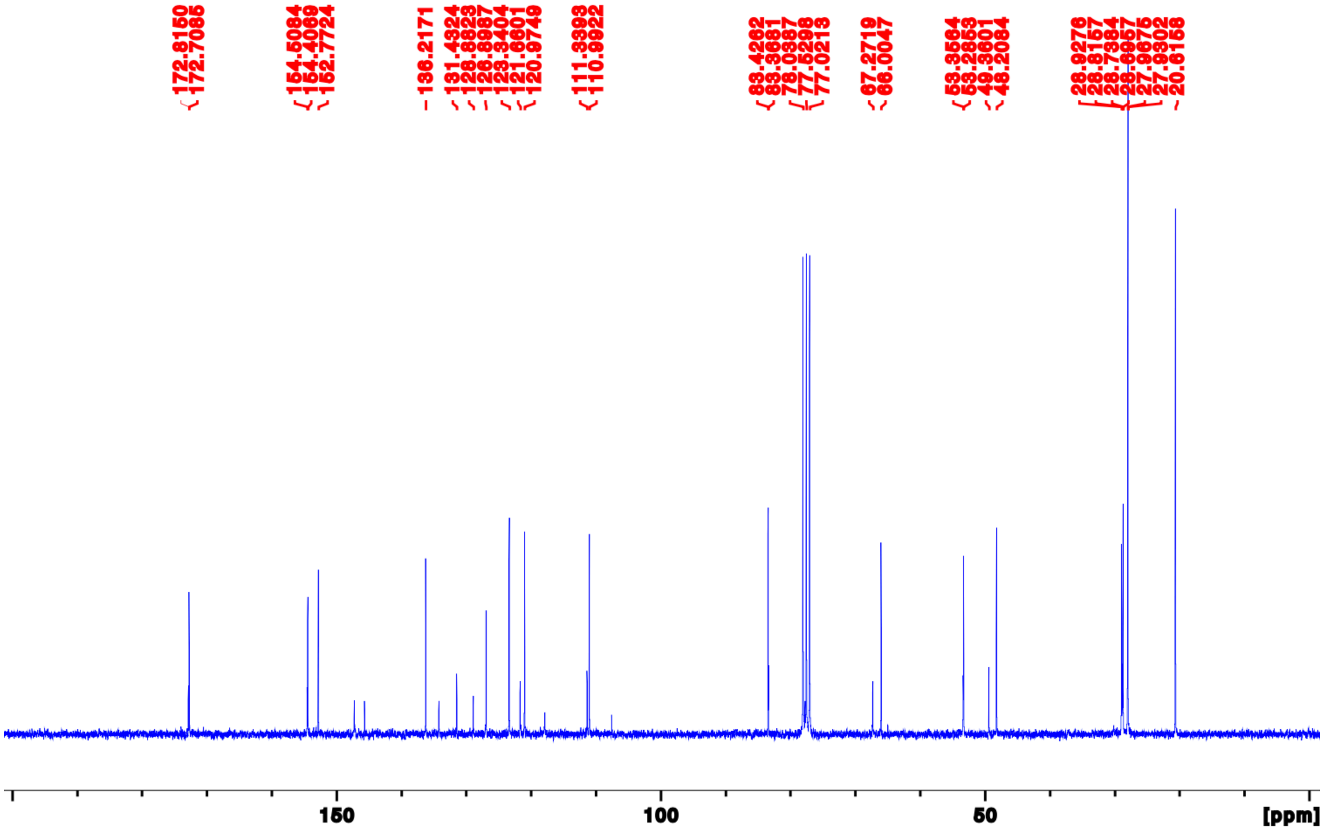

3-(3-Bromopropoxy)-4-nitrobenzoic acid 15

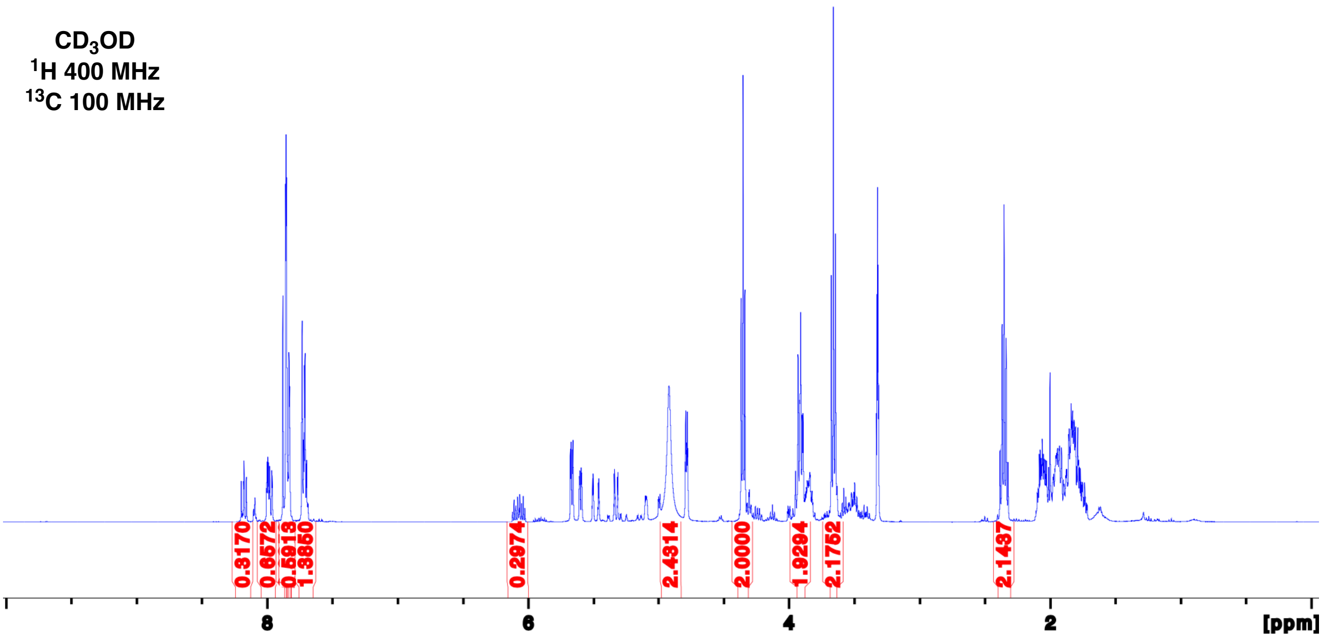

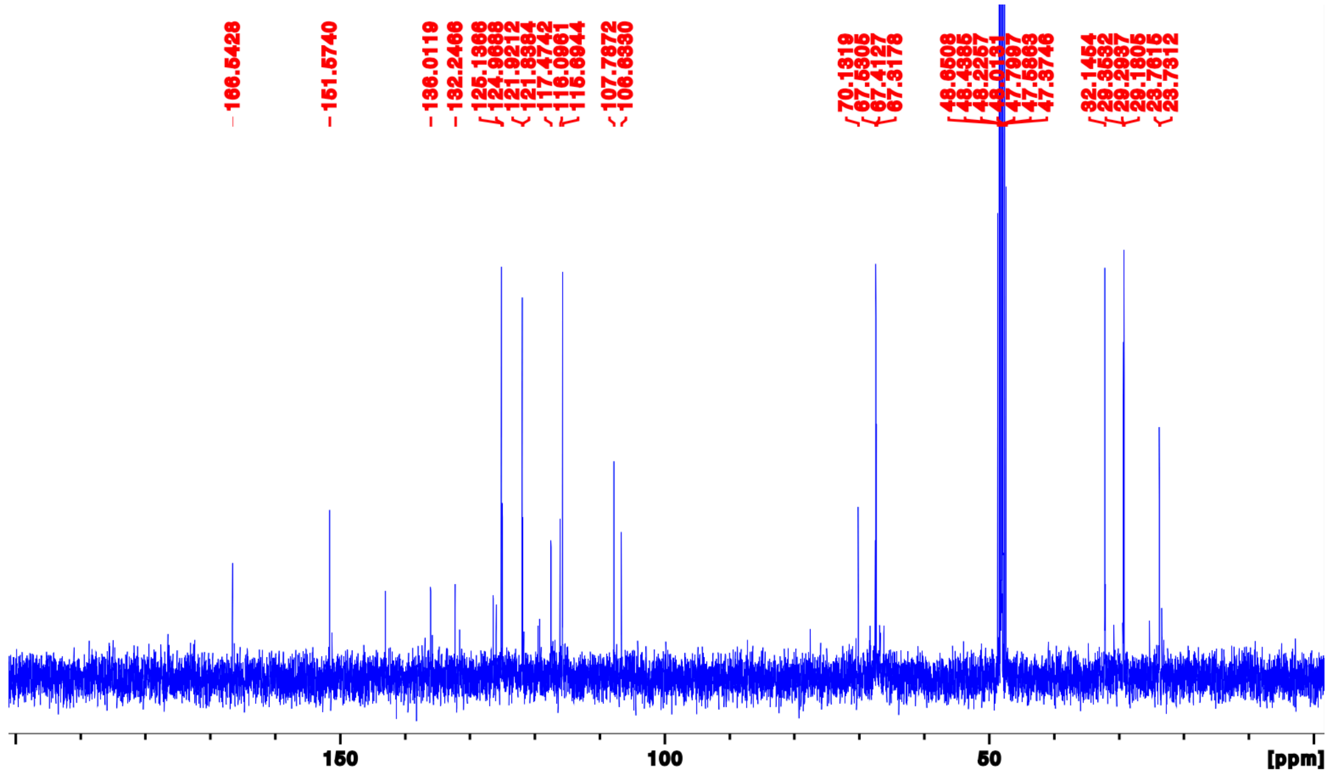

3-(3-(Bis(*tert*-butoxycarbonyl)amino)propoxy-4-nitrobenzoic acid 16

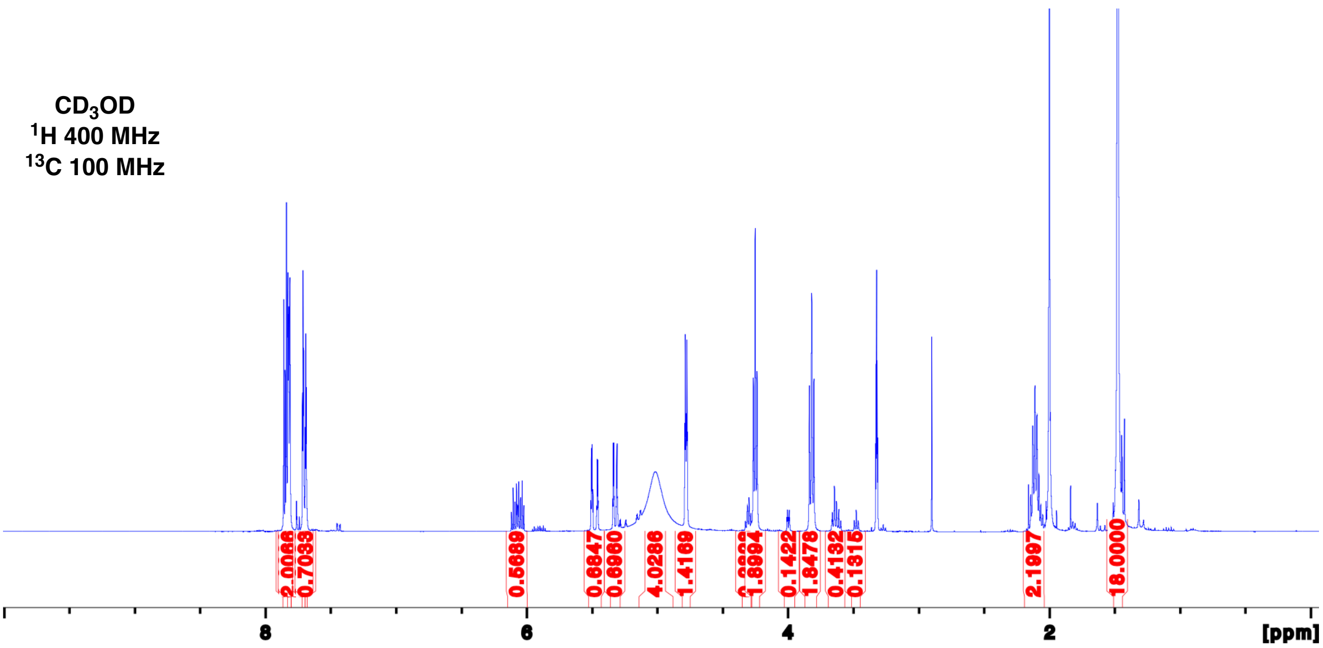

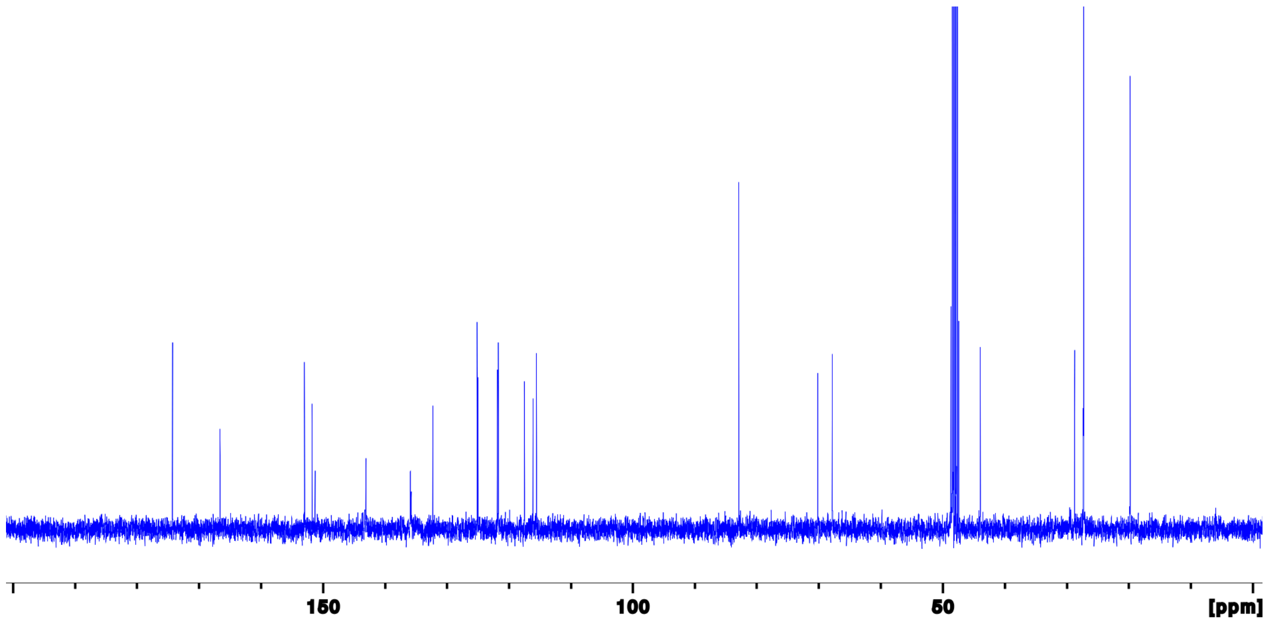

3-(3-(Bis(*tert*-butoxycarbonyl)amino)propoxy- *N*-isobutyl-4-nitrobenzamide 3

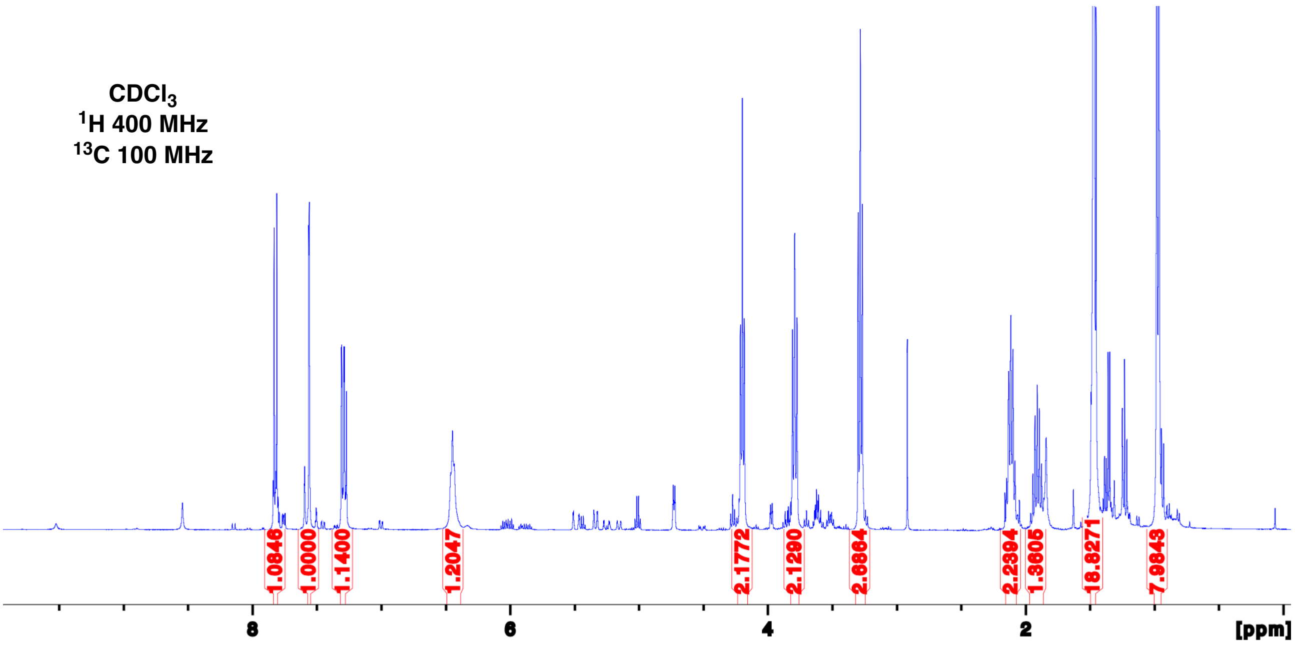

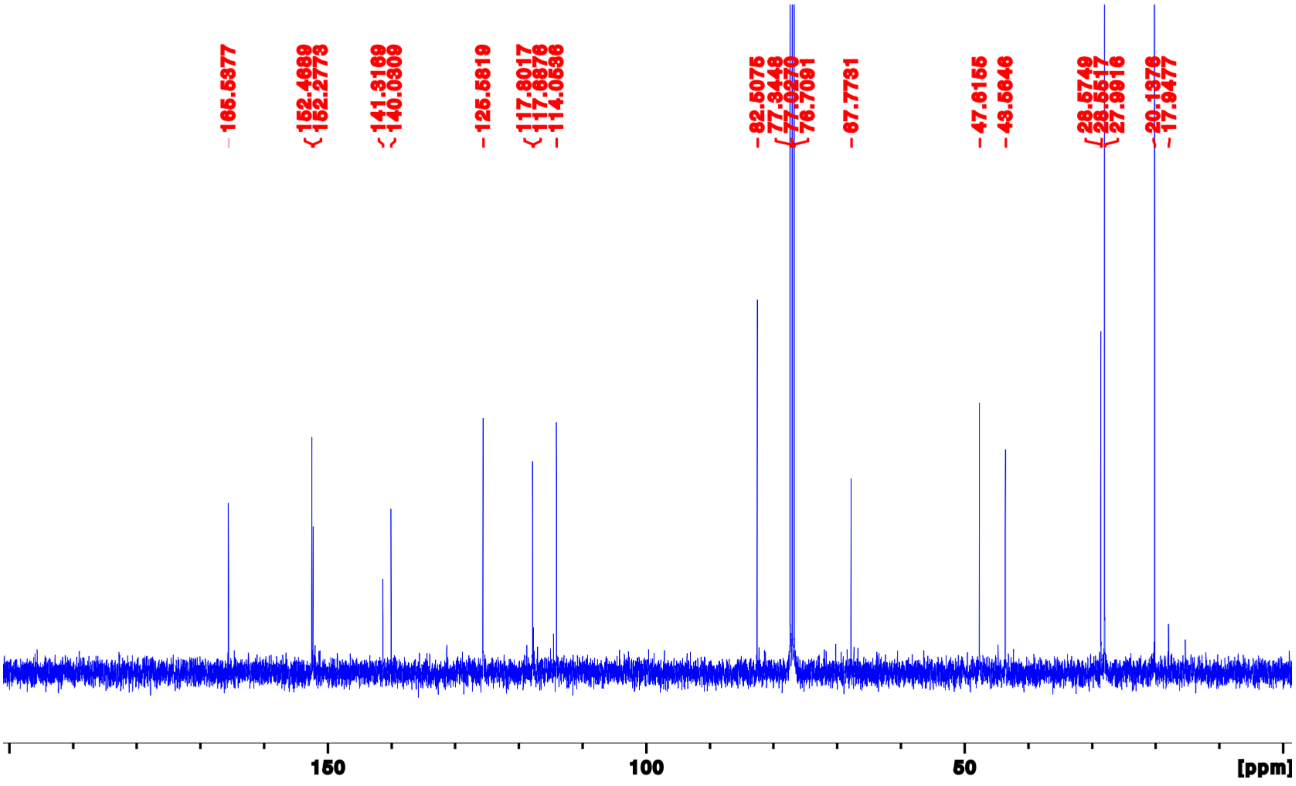

***tert*-Butyl(3-(3-azidopropoxy)-4-(3-(3-(3-(bis(*tert*-butoxycarbonyl)amino)propoxy)-4-nitrobenzoyl)-3-isobutylureido)benzoyl)(isobutyl)carbamate 5**

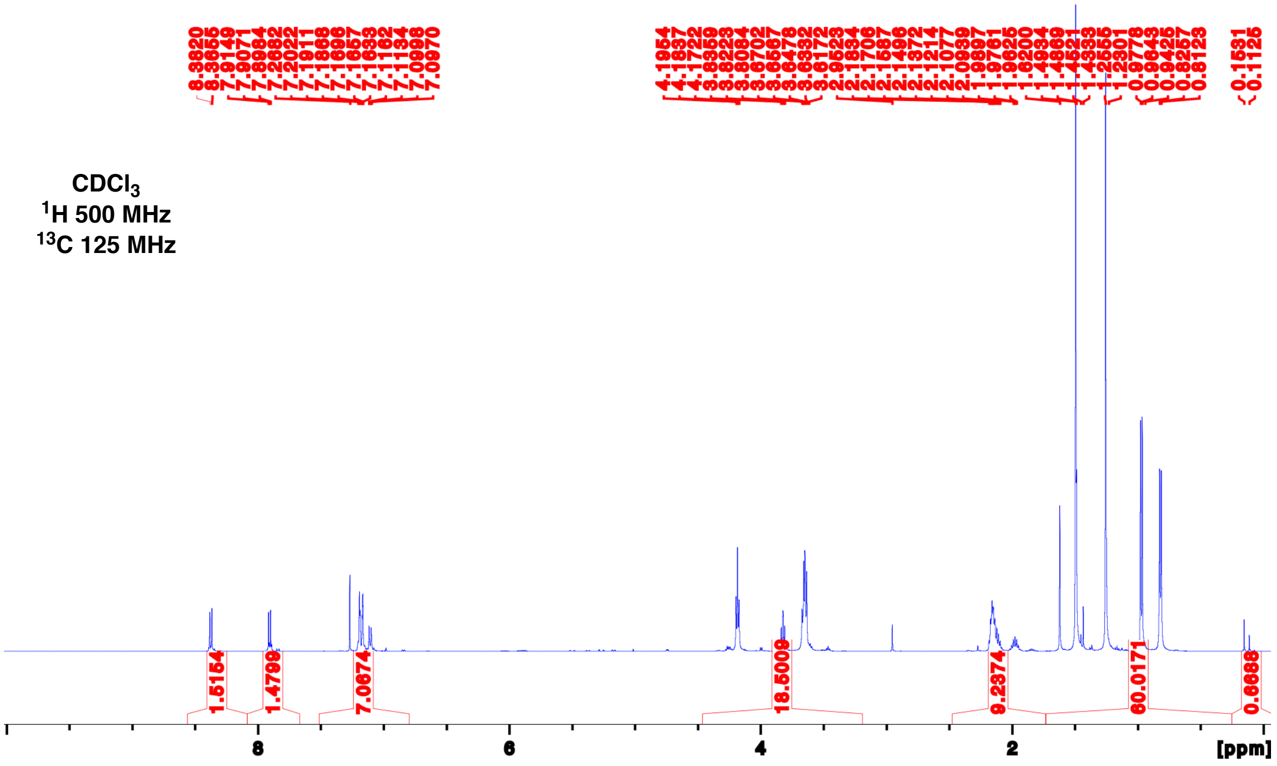

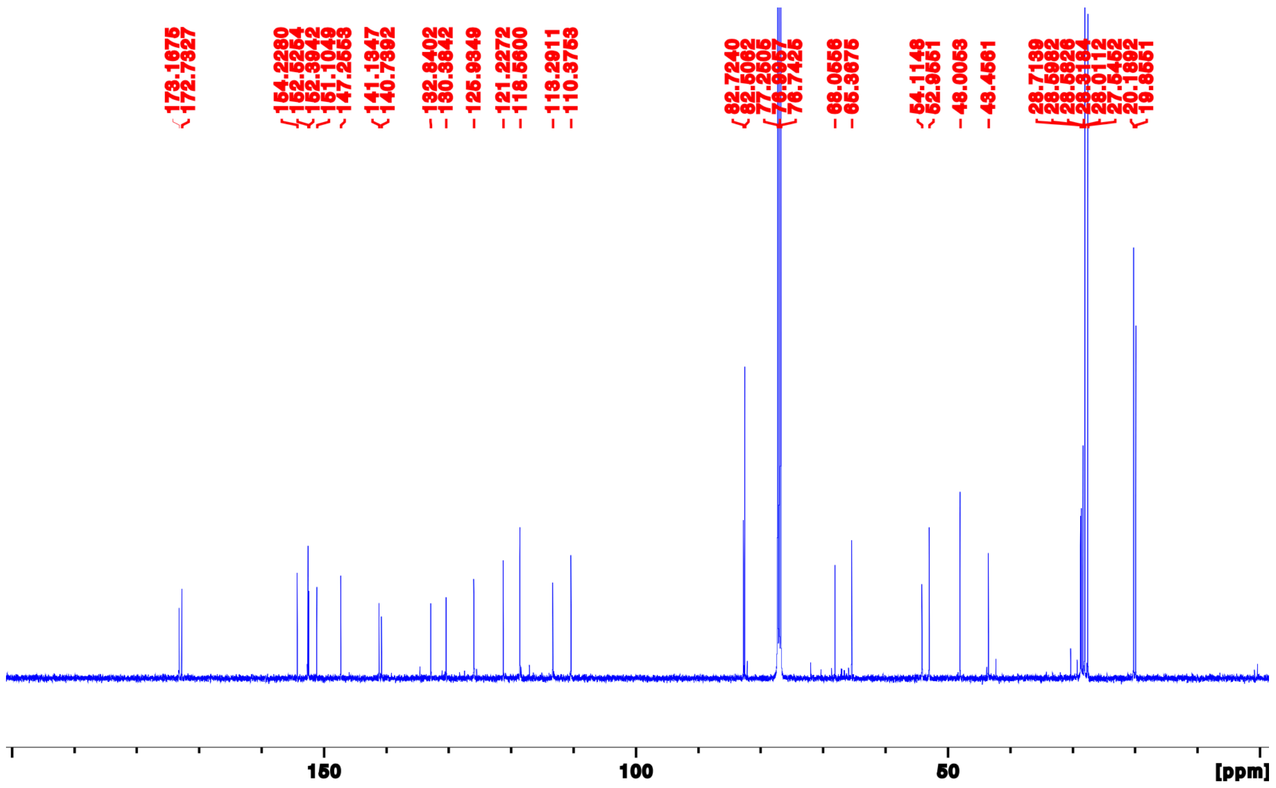

***tert*-Butyl(4-(3-(4-amino-3-(3-(bis(*tert*-butoxycarbonyl)amino)propoxy)benzoyl)-3-isobutylureido)-3-(3-((2,2,10,10-tetramethyl-4,8-dioxo-3,9-dioxa-5,7-diazaundecan-6-yl)amino)propoxy)benzoyl)(isobutyl)carbamate 17**

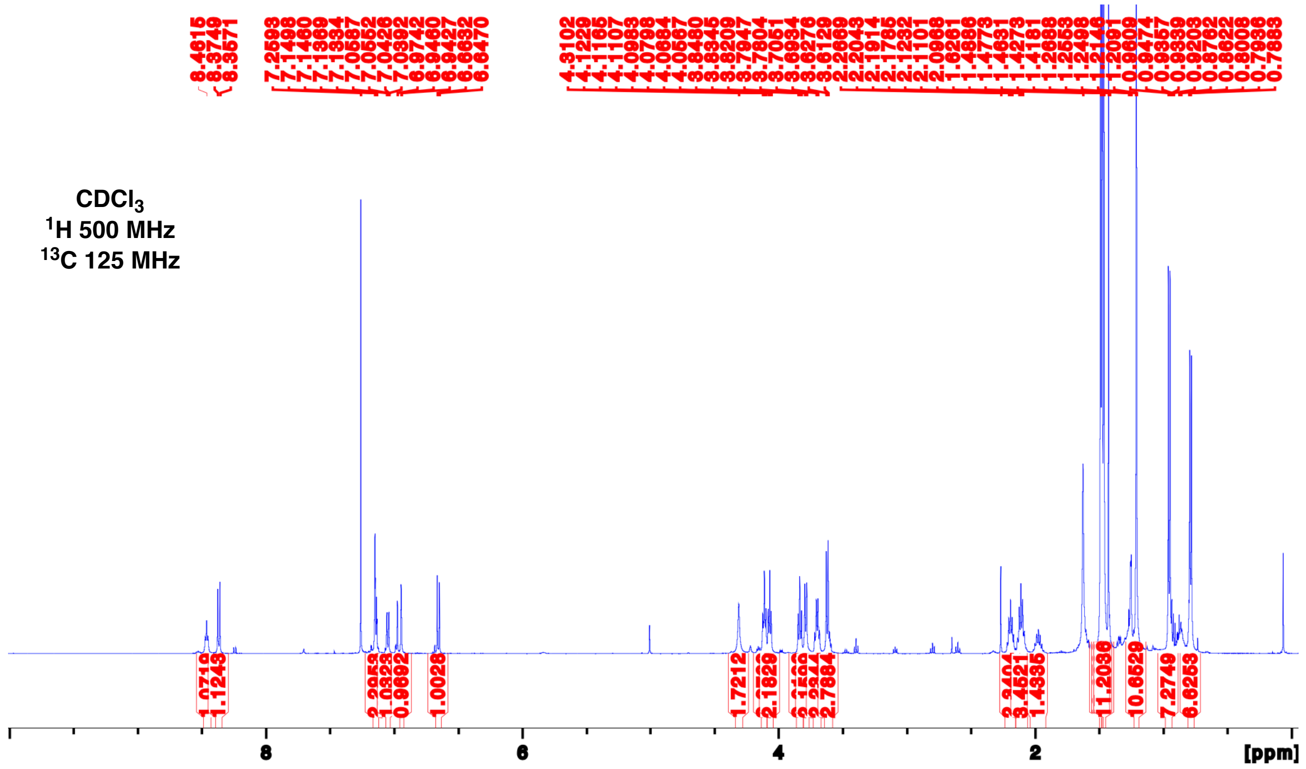

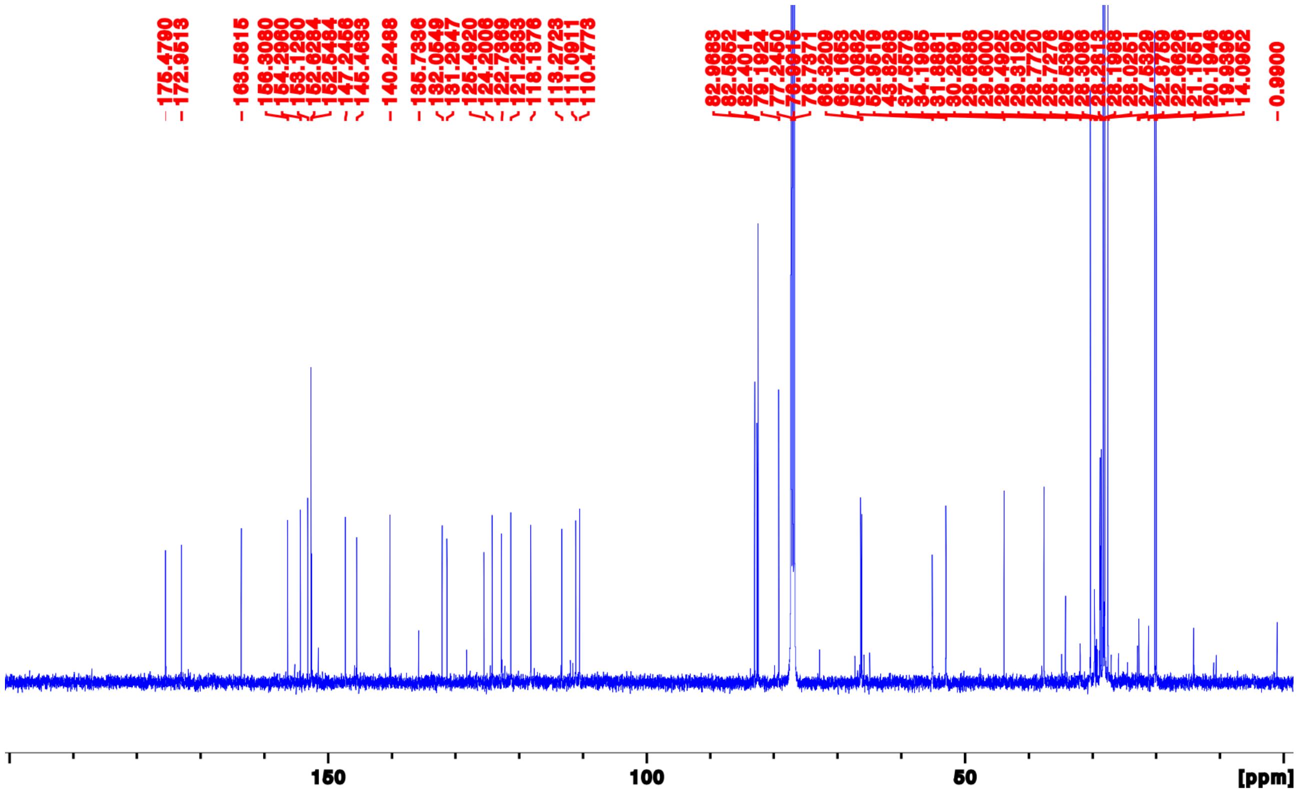

3-(2-Amino-5-(((2-(3-((amino(iminio)methyl)amino)propoxy)-4-(isobutylcarbamoyl)phenyl)carbamoyl)(isobutyl)carbamoyl)phenoxy)propan-1-aminium•2 TFA 1

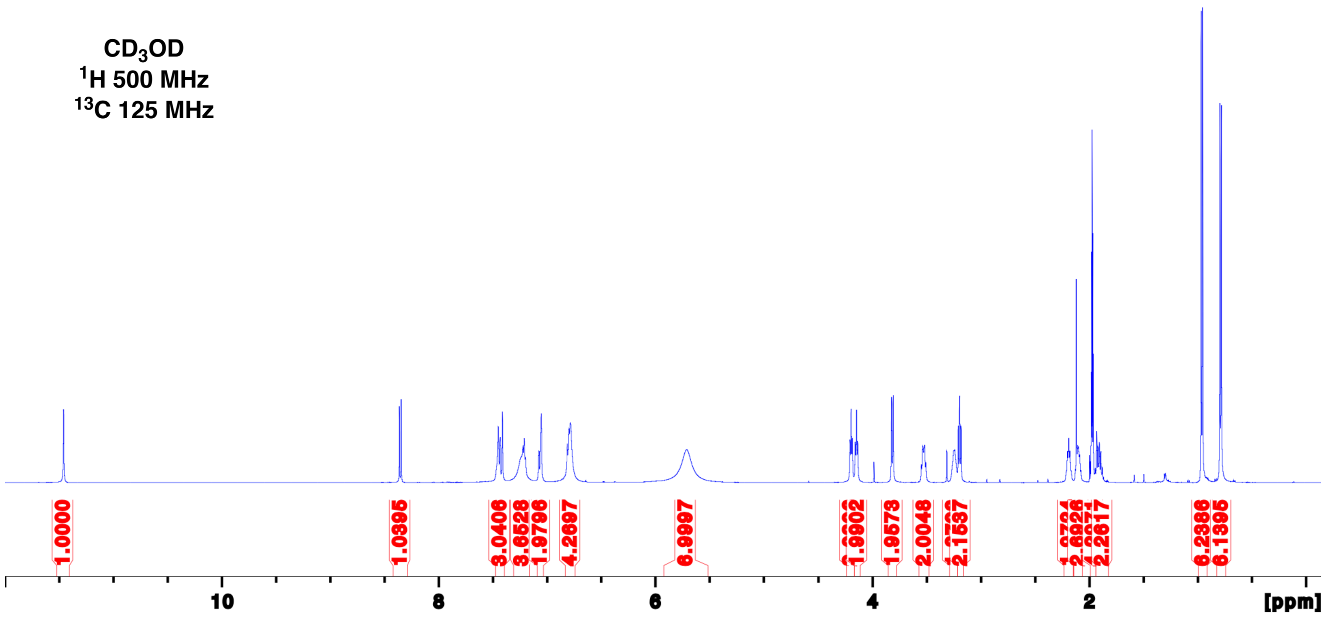

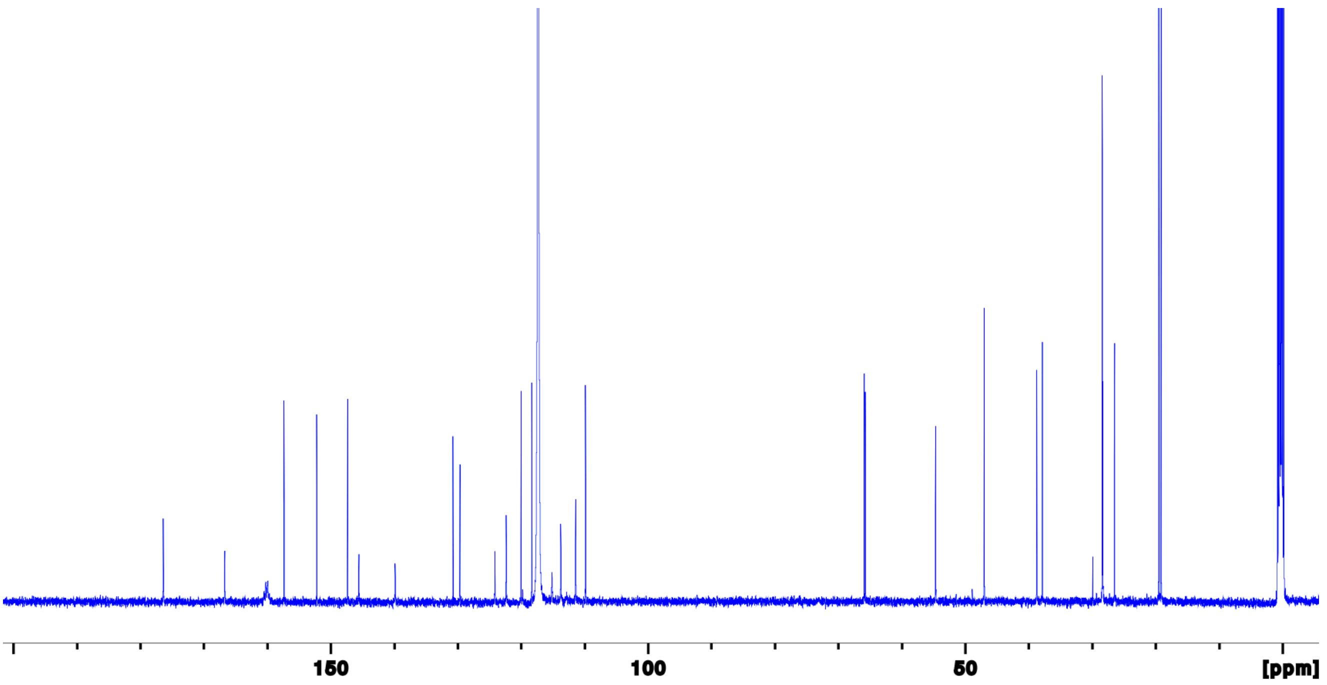

tert-Butyl 3-(3-azidopropoxy)-4-nitrobenzoate 18

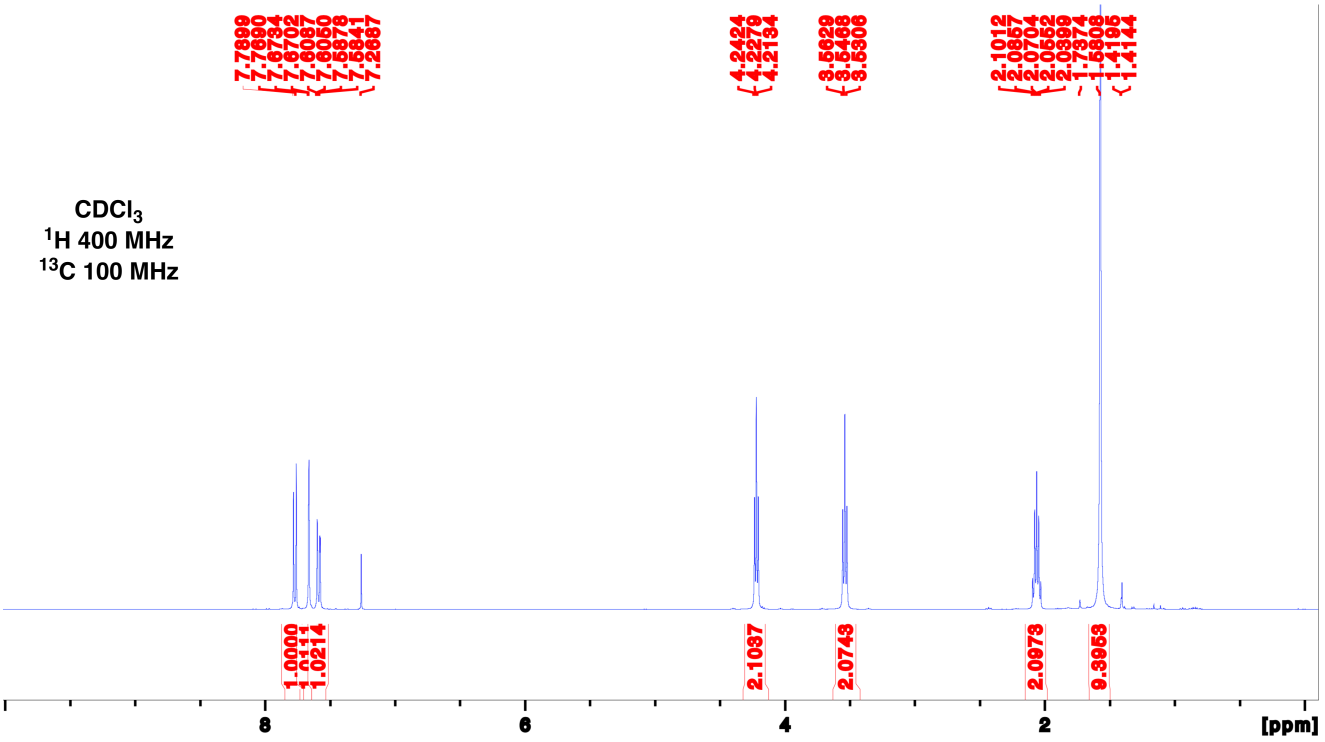

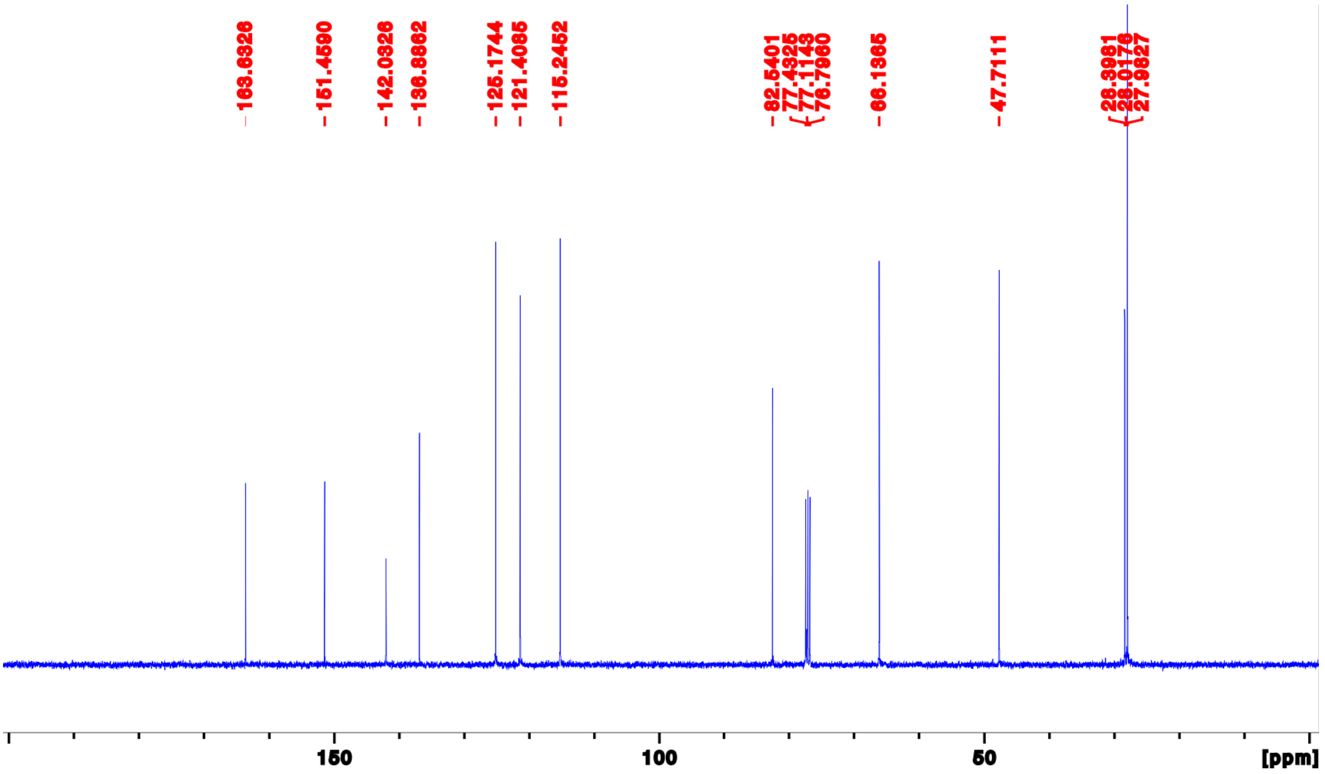

tert-Butyl 4-amino-3-(3-azidopropoxy)benzoate 7

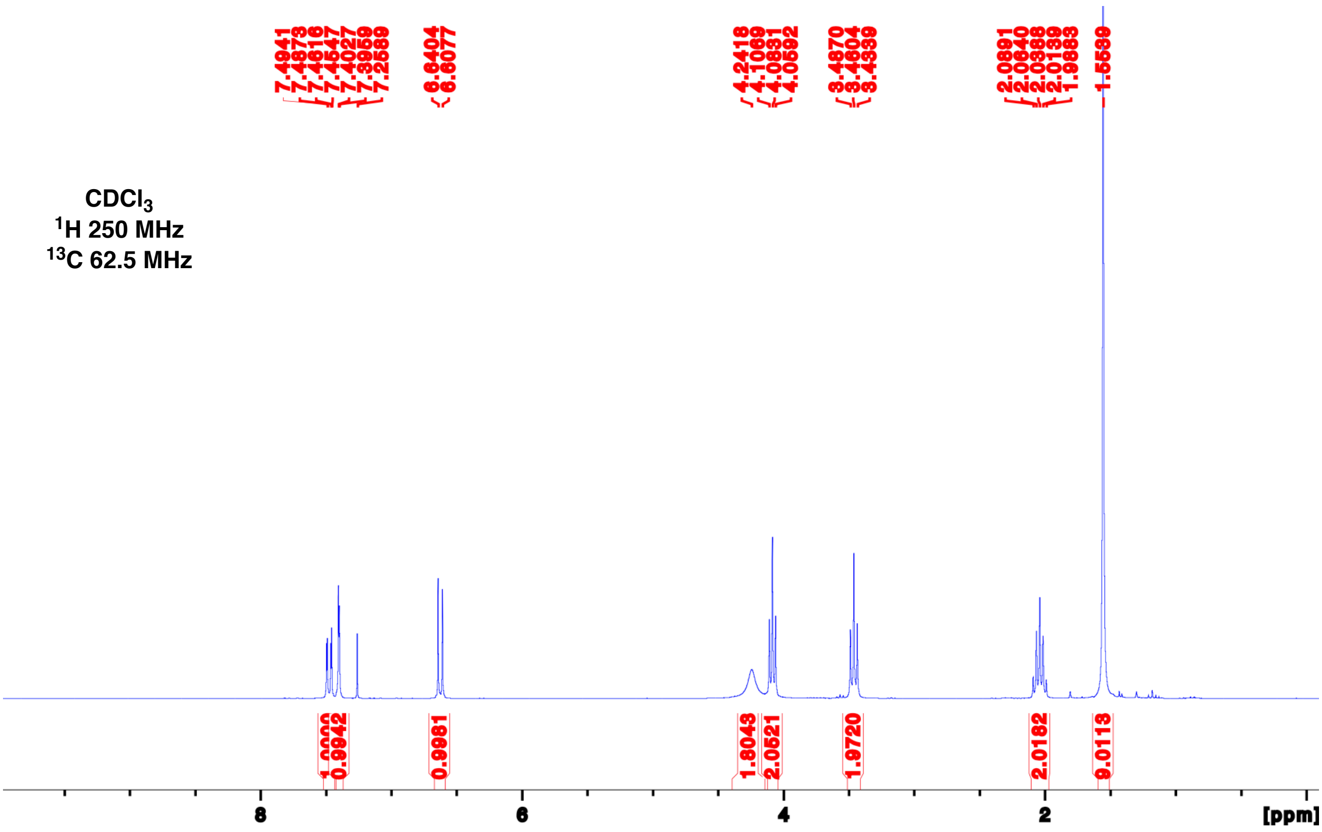

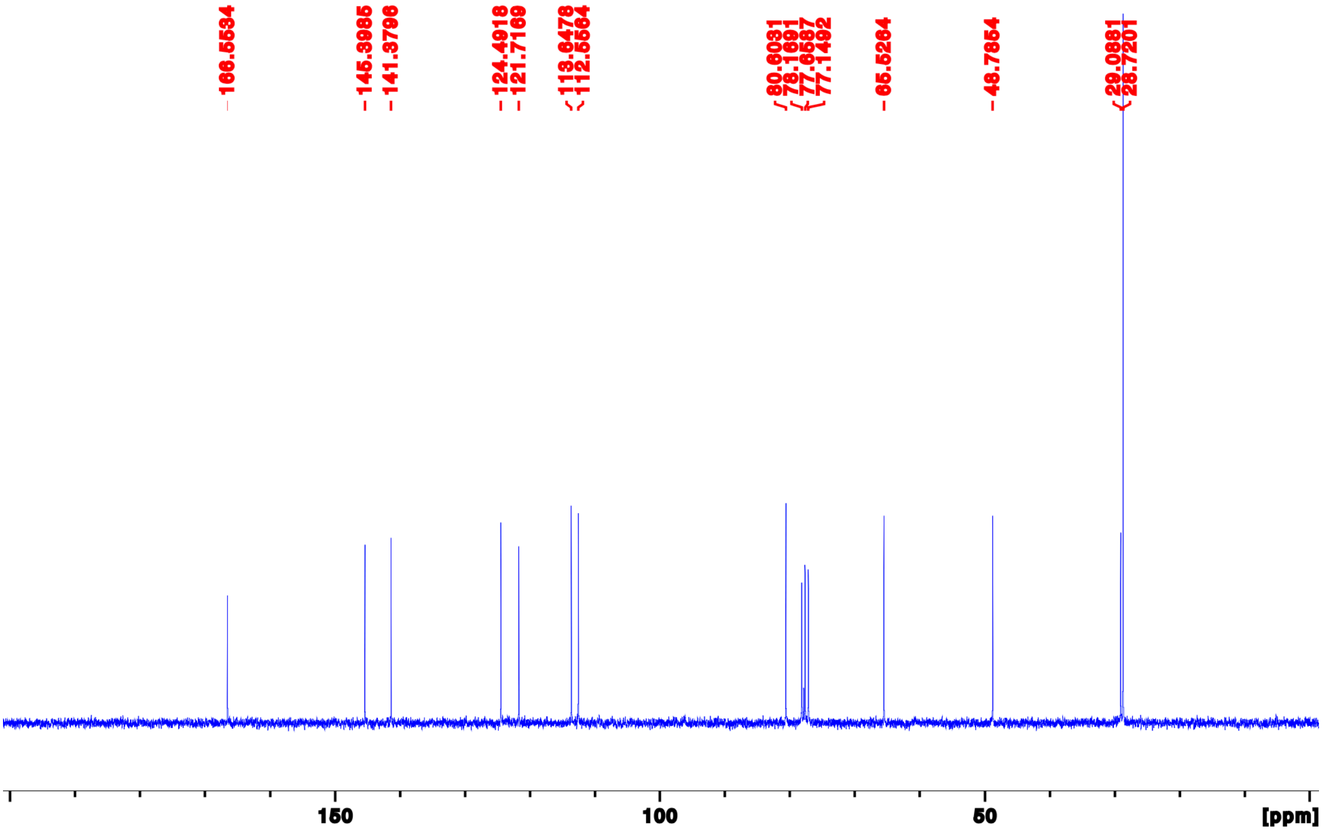

***tert*-Butyl 3-(3-azidopropoxy)-4-(3-(3-((*tert*-butoxycarbonyl)amino)propoxy)-4-nitrobenzamido) benzoate 9**

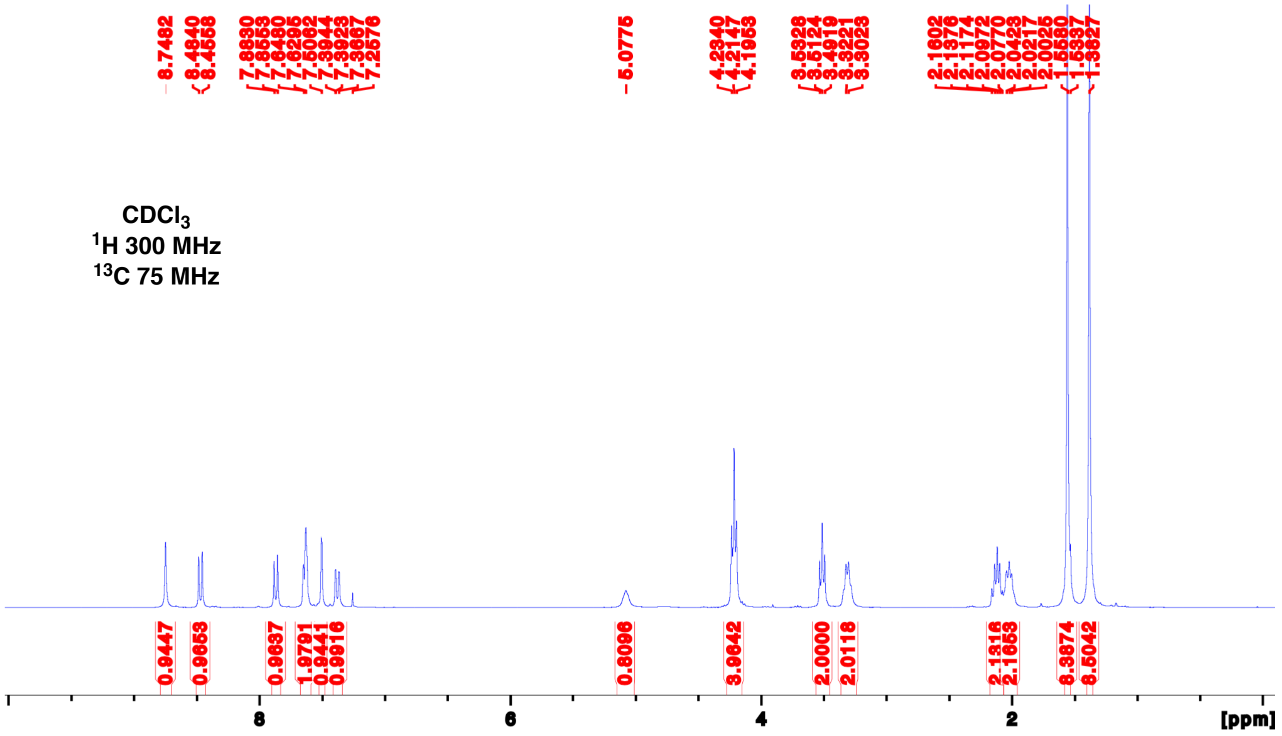

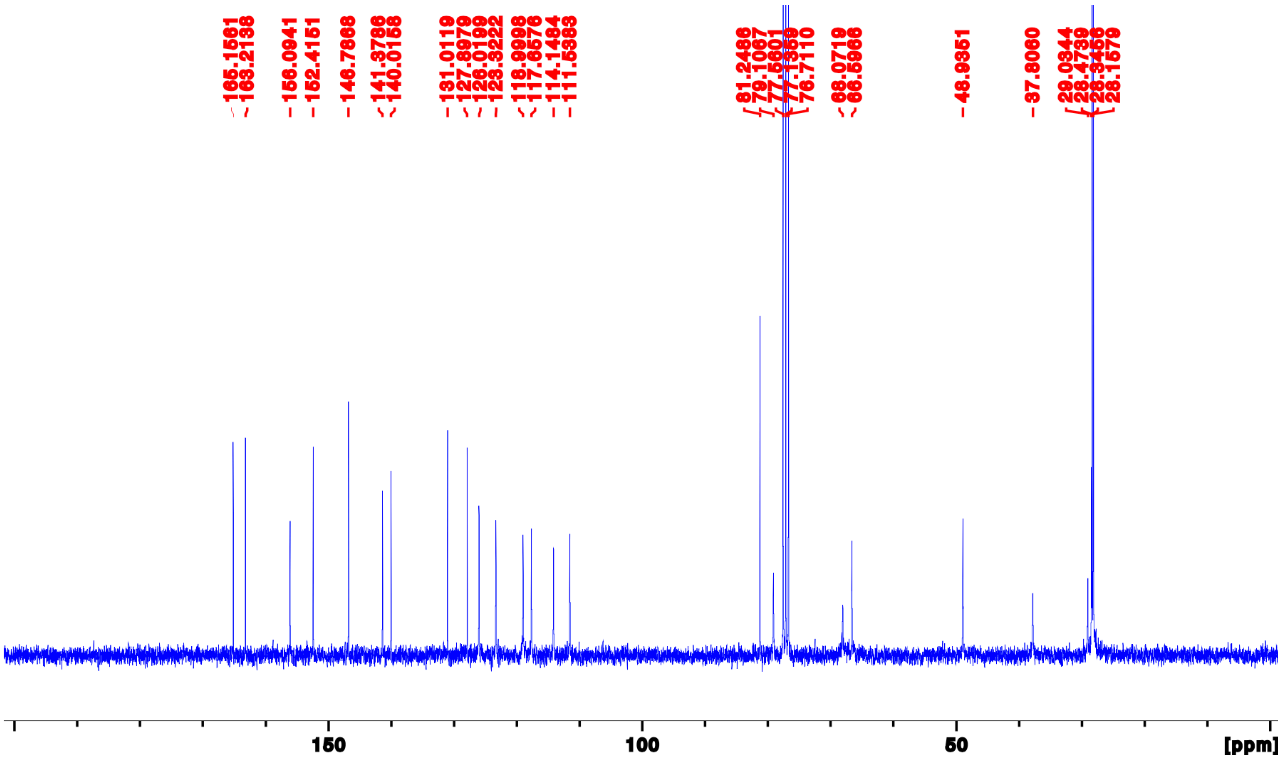

tert-Butyl 4-(4-amino-3-(3-((*tert*-butoxycarbonyl)amino)propoxy)benzamido)-3-(3-aminopropoxy)benzoate 19

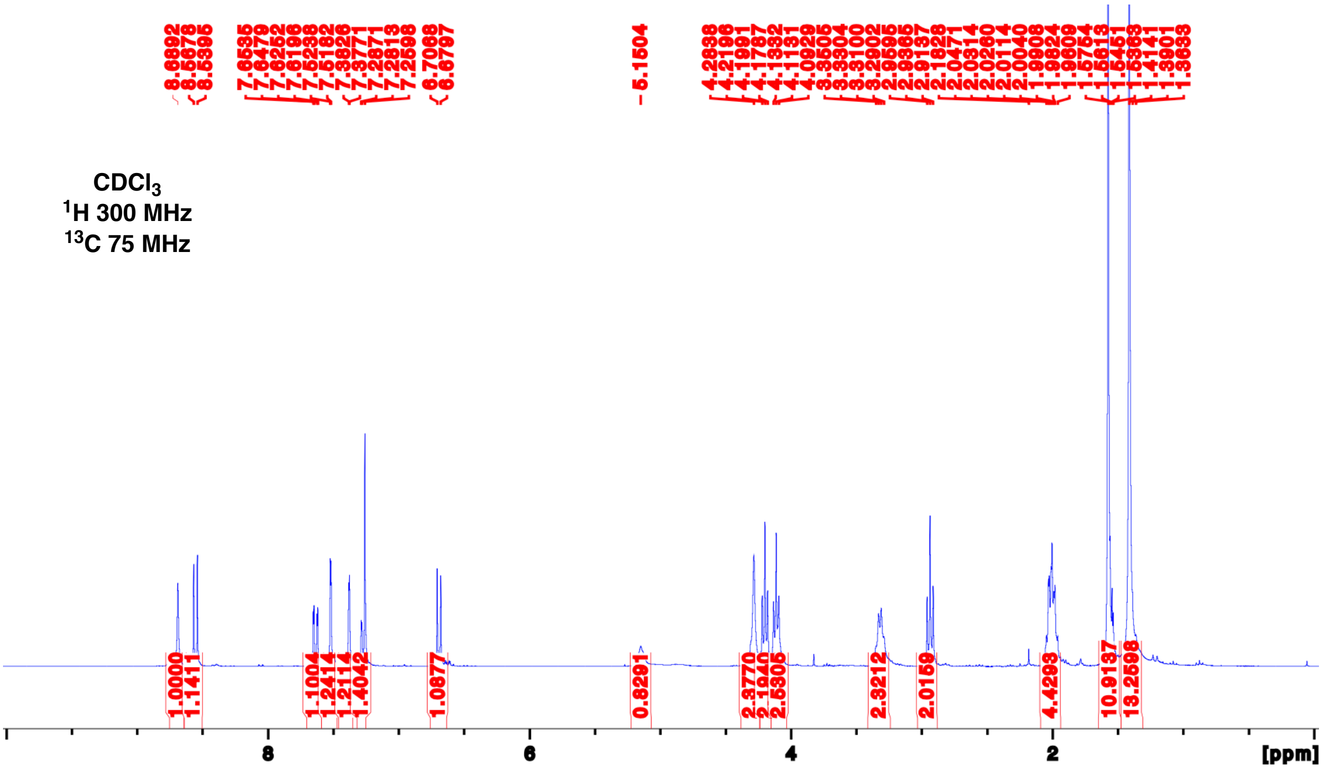

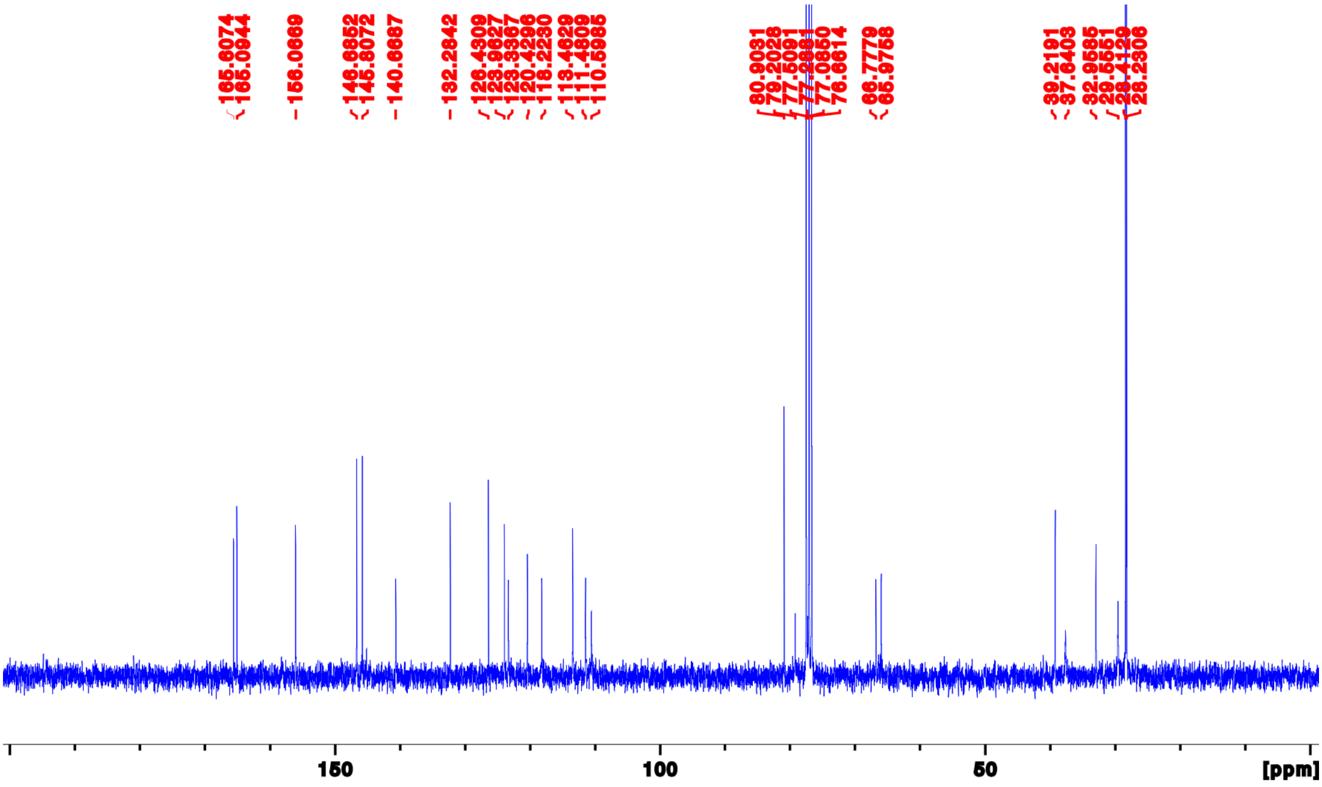

***tert*-Butyl 4-(4-amino-3-(3-((*tert*-butoxycarbonyl)amino)propoxy)benzamido)-3-(3-(2,3-bis(*tert*-butoxycarbonyl)guanidino)propoxy)benzoate 20**

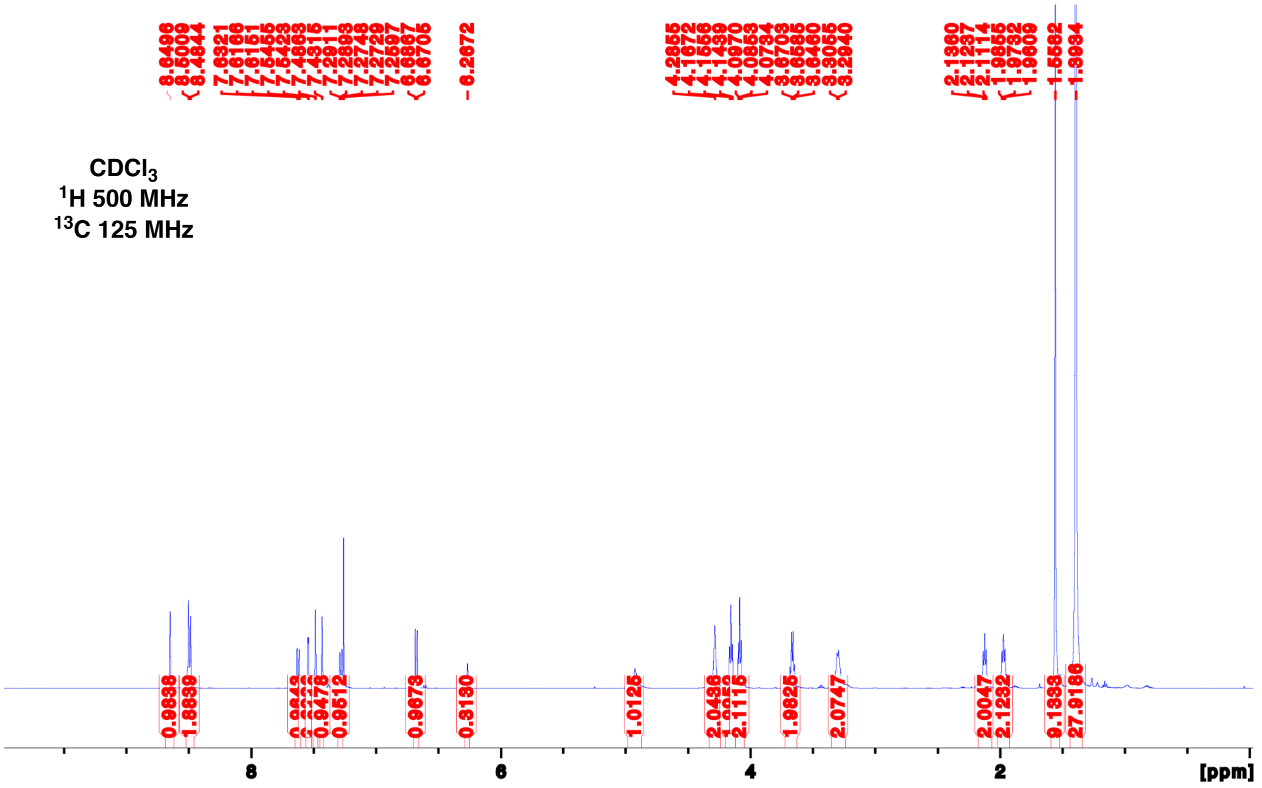

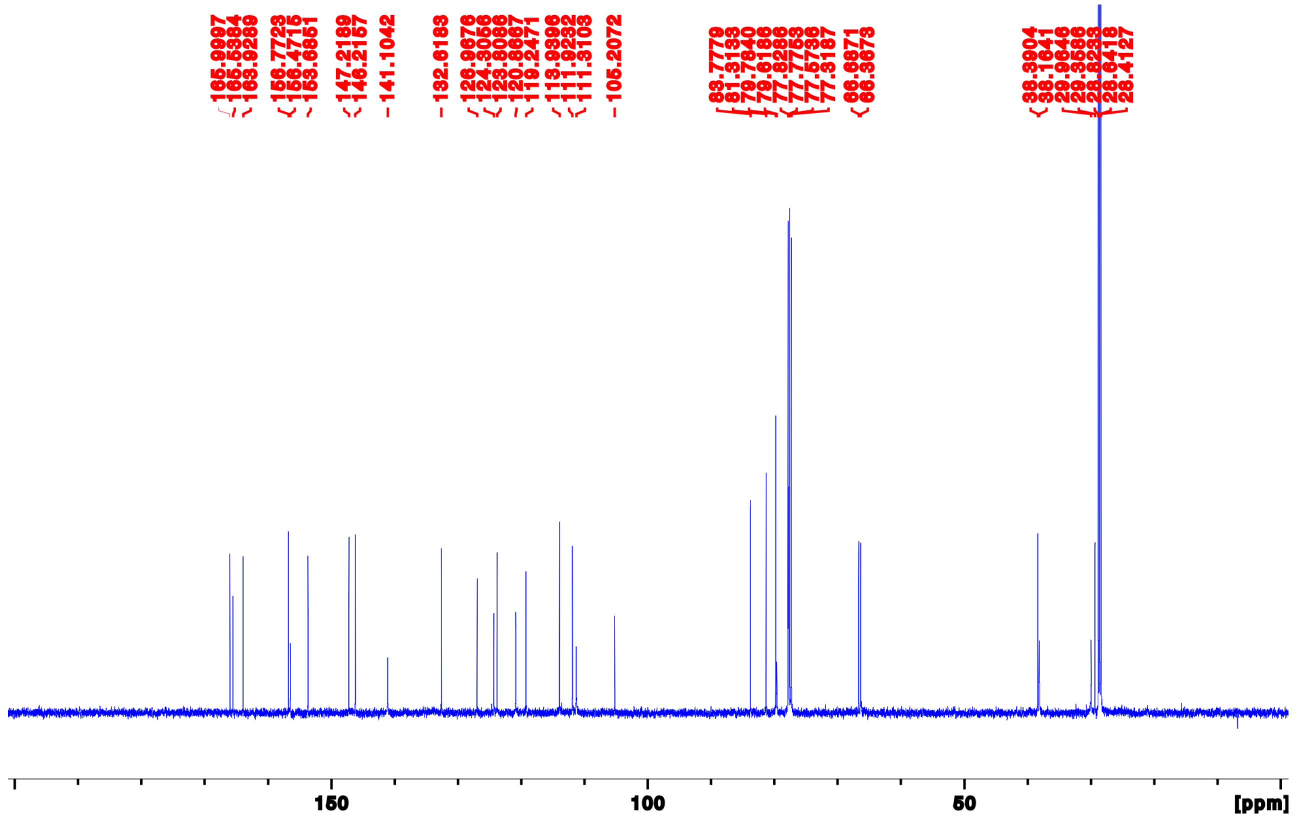

4-(4-Amino-3-(3-aminopropoxy)benzamido)-3-(3-guanidinopropoxy)benzoic acid•2TFA 2

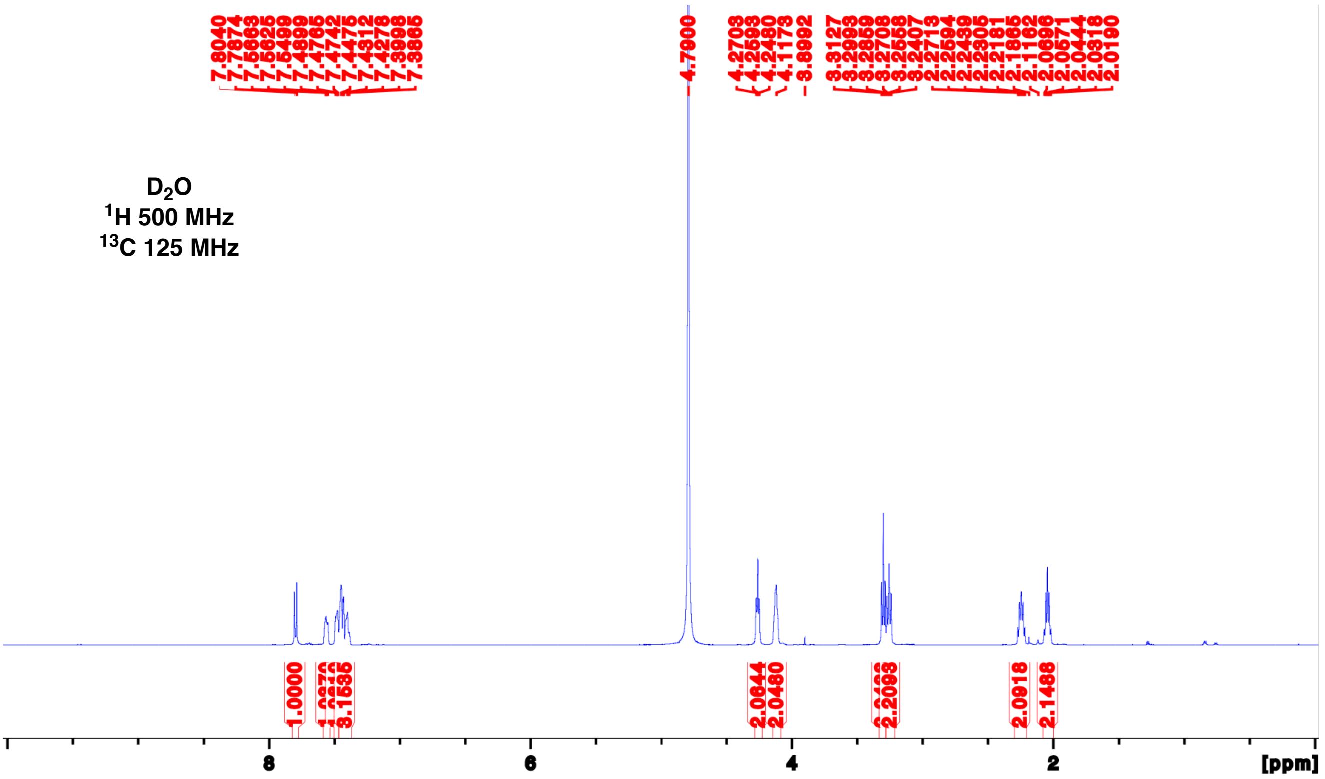

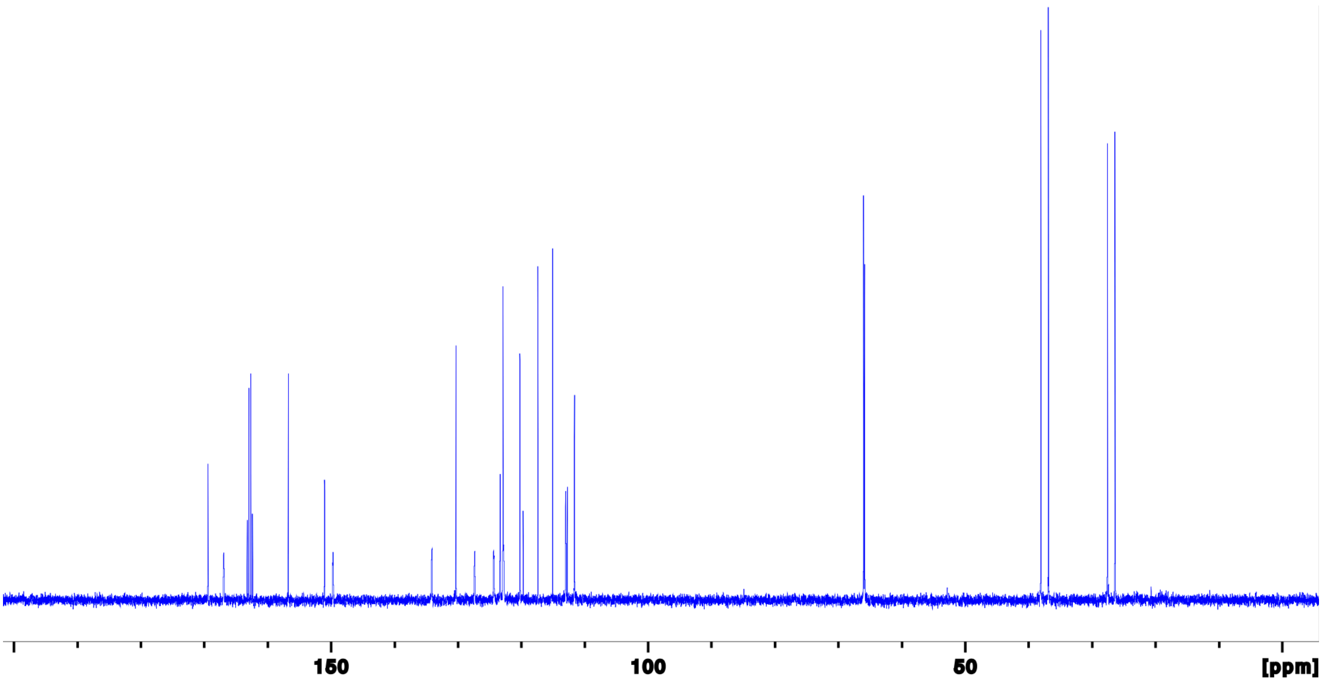

4. Protein Expression and Purification

We have previously described pGEX-vectors for human Grb2SH3C.[5] For protein expression, bacteria were grown in terrific broth with 100 mg/ml ampicillin. Expression was induced at an OD_{600} of ca. 0.8 with 0.05 mM IPTG overnight at 18 ˚C. Bacteria were then sedimented by centrifugation. Chilled bacterial pellets were lysed in cold TPE (1% [v/v] Triton X-100, PBS [pH 7.4], 100 mM EDTA, 10 mg/ml aprotinin, 0.7 mg/ml pepstatin A, 0.5 mg/ml leupeptin, and 5 mg/ml antipain) and sonicated, and the lysate was clarified by centrifugation at 20,000 x g for 1 h at 4 ˚C. GST-fusion protein was purified by co-incubating the cleared supernatant with GSH-sepharose beads overnight at 4 ˚C on a nutator. Beads were then washed extensively with 50 mM TrisHCl (pH 7.5), 100 mM EDTA, and 0.1% (v/v) Tween 20. Bound GST-fusion protein was eluted with 100 mM GSH pH-adjusted to pH 7.5 with TrisHCl (pH 8.8), and the eluate was dialyzed against 5 mM TrisHCl (pH 7.5). The integrity of the dialysed protein was analysed by SDS-PAGE and Coomassie Blue staining, and the protein concentration was assayed by the Bradford method. Purified GST-SH3 fusion protein was snap frozen in aliquots and stored at -80 ˚C until further use.

For Grb2 SH3C fusion protein without detergent and GST-tag, chilled bacterial pellets were dissolved in 50 mM HEPES (pH 7.5) with 50 mM NaCl, 0.2 mM PMSF, and Complete™ protease inhibitors (Roche), then sonicated and centrifuged to remove debris as before. Cleared lysates were incubated with GSH-sepharose, washed extensively, and cleaved with 3C protease (PreScission protease, GE Healthcare) in 50 mM TrisHCl (pH 7.0), 150 mM NaCl, 1 mM EDTA to remove the GST-tag. SH3C protein was eluted, the solution was cleared by centrifugation for 10 min at 20,000 x g and the SH3 domain was further purified by size exclusion FPLC on a HiLoad 16/60 Superdex S75 column equilibrated with 50 mM HEPES (pH 7.5) and 150 mM NaCl. Purified protein was analyzed by SDS-PAGE and Coomassie Blue staining and then was concentrated (Vivaspin VS0611; Vivascience). Its concentration was quantified by UV absorption at 280 nm.

5. Surface Plasmon Resonance

SPR was performed using a Biacore T200 optical biosensor. A fresh CM5 chip was docked to the instrument and hydrated with duplicate 12 s injections of 50 mM NaOH, 10 mM Glycine•HCl pH 1.5, and 0.1 % SDS at 50 μL.min^{-1} flow rate. The flow cells were then normalized with 70 % glycerol using the default normalization wizard. The chip was primed into HBS-N running buffer (10 mM Hepes pH 7.4, 150 mM NaCl), and the flow cells activated with a 1:1 mixture of 0.4 M EDC and 0.1 M NHS for 10 minutes at 10 μL.min^{-1} and 25 °C. ^{15}N labeled Grb2 SH3C (100 μM in pH 4.0 acetate buffer) was coupled to the surface and covalently bound to a level of 1100 RU. After coupling, all flow cell surfaces were deactivated with 4 x 30 s injections of a 1:1 mixture of 1.0 M ethanolamine and running buffer. The chip was subsequently primed twice into the experimental running buffer (HBS-N buffer + 0.01 % Tween 20). Molecules were tested at a flow rate of 40 μL.min^{-1} at 25 °C, with a data collection rate of 10 Hz. The data were analyzed using standard double referencing procedures by subtracting the response from the protein-free cell and the response from a blank injection of running buffer from the response from the SH3C flow cell. Data was collected according to the general SPR procedure and fitted to a one-to-one binding model using nonlinear regression analysis in GraphPad Prism software.

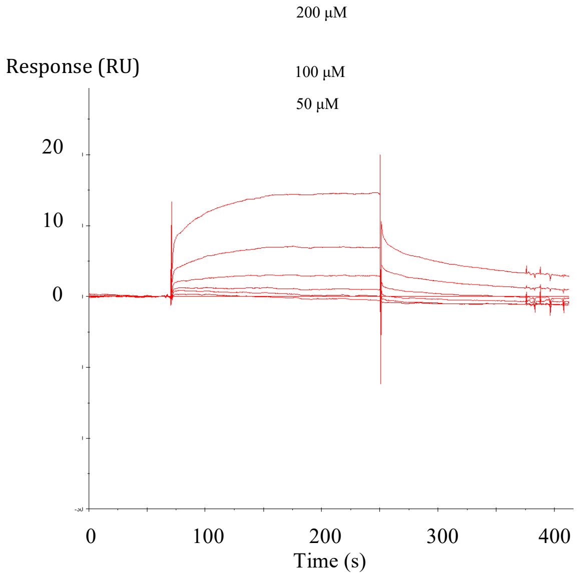

Figure S-2: SPR binding of α-helix mimic **2** binding to ^{15}N-labeled Grb2 SH3C. Mimic concentrations are a two-fold dilution series from 200 μM (highest response) to 6.25 μM (lowest non-zero response). The lowest mimic concentrations yield sensograms that are too similar to be analysed. The maximal binding response at each concentration does not fit to a 1:1 binding model, and hence the dissociation constant for this mimic is undefined.

6. Protein NMR

All spectra were collected using a Bruker AVII 700 spectrometer (fitted with an inverse TCI cryoprobe optimized for ^{1}H observation and running Topspin 2.1 software; Bruker, Germany). Experiments were performed at 25 ˚C. Resonances are reported in ppm relative to D_2O (δ_H = 4.72 ppm). The instrument was locked to the deuterium signal, and the water signal was suppressed by presaturating its resonance. Grb2 SH3C was uniformly ^{15}N isotopically labelled and dissolved in phosphate buffer (90:10 H_2O:D_2O, 35 mM phosphate pH 7.2 + 45 mM NaCl + 3.0 mM DTT). A 3 mM NMR tube was charged with 160 µL of sample and the ^{15}N HSQC spectrum recorded. Resonances were compared to the literature values.[6] For titration experiments, varying molar equivalents of ligand were added to the protein solution described above. Significance thresholds for shifts are defined as those residues that shift 1.5x greater than the standard deviation for all shifts. Data was analyzed using Bruker's Topspin 2.1 software.

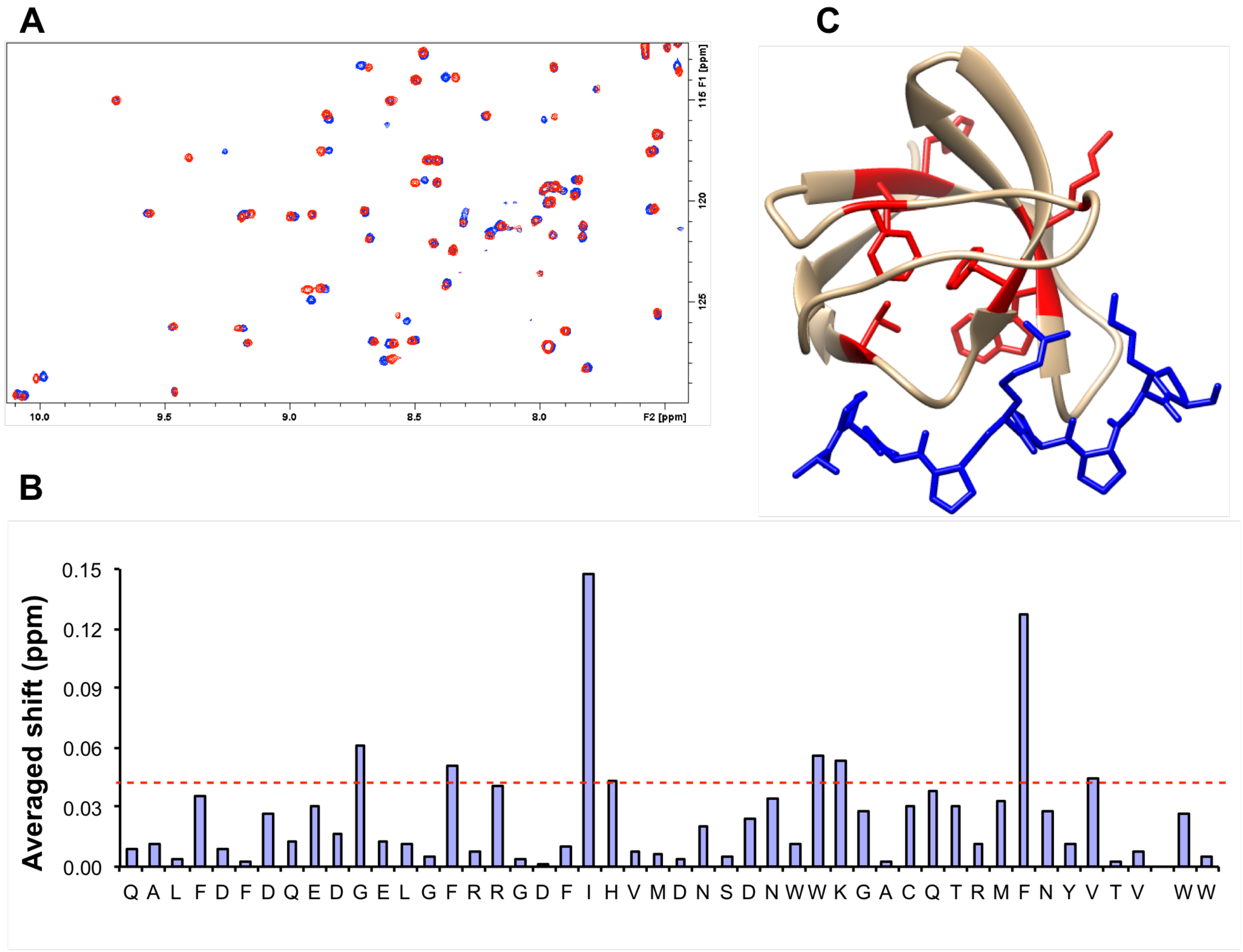

Figure S-3: NMR analysis of **2**/Grb2 interaction: a) ^{1}H-^{15}N HSQC spectrum of uniformly ^{15}N-labeled Grb2 in the absence (red) and presence (blue) of 20 equivalents of molecule **2**; b) Chemical shift changes for Grb2. The significance threshold of 0.043 ppm is indicated by a red dashed line. c) Molecule **2** overlayed on the structure of Grb2 (PDB 2W0Z), with residues found to shift significantly in the HSQC experiment shaded red. The analysis indicates few surface residue shifts, and the two largest shifts (Ile25 and Phe47) are completely solvent-excluded, with their sidechains pointing into the conserved β-sandwich of the protein. This indicates that binding results in protein unfolding rather than specific surface binding.

7. Supplemental References

[1] Spartan; Schrödinger, LLC, New York, NY, 2025.
[2] In *The PyMOL Molecular Graphics System, Schrödinger, LLC.*
[3] J. G. Badiang, J. Aubé, *J. Org. Chem.* **1996**, *61*, 2484–2487.
[4] A. Shaginian, L. R. Whitby, S. Hong, I. Hwang, B. Farooqi, M. Searcey, J. Chen, P. K. Vogt, D. L. Boger, *J. Am. Chem. Soc.* **2009**, *131*, 5564–5572.
[5] M. Harkiolaki, T. Tsirka, M. Lewitzky, P. C. Simister, D. Joshi, L. E. Bird, E. Y. Jones, N. O'Reilly, S. M. Feller, *Structure* **2009**, *17*, 809–822.
[6] C.-C. Lin, F. A. Melo, R. Ghosh, K. M. Suen, L. J. Stagg, J. Kirkpatrick, S. T. Arold, Z. Ahmed, J. E. Ladbury, *Cell* **2012**, *149*, 1514–1524.
